# Reactive Oxygen Species‐Responsive Polymeric Prodrug Nanoparticles for Selective and Effective Treatment of Inflammatory Diseases

**DOI:** 10.1002/adhm.202301394

**Published:** 2023-08-17

**Authors:** Yaming Zhang, Lu Liu, Tianyi Wang, Cong Mao, Pengfei Shan, Chak Sing Lau, Zhongyu Li, Weisheng Guo, Weiping Wang

**Affiliations:** ^1^ State Key Laboratory of Pharmaceutical Biotechnology & Dr. Li Dak‐Sum Research Centre & Department of Pharmacology and Pharmacy Li Ka Shing Faculty of Medicine The University of Hong Kong Hong Kong SAR China; ^2^ Department of Minimally Invasive Interventional Radiology State Key Laboratory of Respiratory Disease School of Biomedical Engineering & The Second Affiliated Hospital Guangzhou Medical University Guangzhou 510260 China; ^3^ College of Chemistry and Materials Engineering Wenzhou University Wenzhou 325027 China; ^4^ Department of Medicine Li Ka Shing Faculty of Medicine The University of Hong Kong Hong Kong SAR China

**Keywords:** cinnamaldehyde, polymeric prodrugs, rheumatoid arthritis, ROS‐responsive nanoparticles, ulcerative colitis

## Abstract

It is challenging to manage inflammatory diseases using traditional anti‐inflammatory drugs due to their limited efficacy and systemic side effects, which are a result of their lack of selectivity, poor stability, and low solubility. Herein, it reports the development of a novel nanoparticle system, called ROS‐CA‐NPs, which is formed using polymer‐cinnamaldehyde (CA) conjugates and is responsive to reactive oxygen species (ROS). ROS‐CA‐NPs exhibit excellent drug stability, tissue selectivity, and controlled drug release upon oxidative stress activation. Using mouse models of chronic rheumatoid arthritis and acute ulcerative colitis, this study demonstrates that the systemic administration of ROS‐CA‐NPs results in their accumulation at inflamed lesions and leads to greater therapeutic efficacy compared to traditional drugs. Furthermore, ROS‐CA‐NPs present excellent biocompatibility. The findings suggest that ROS‐CA‐NPs have the potential to be developed as safe and effective nanotherapeutic agents for a broad range of inflammatory diseases.

## Graphical abstract

This study develops a simple ROS‐responsive prodrug nanosystem to improve drug performance while reducing unwanted side effects. The high level of ROS at inflamed sites can trigger the cleavage of ROS‐sensitive linker, which can further stimulate the functional group exposure of drugs for the treatment of both acute and chronic inflammatory diseases.

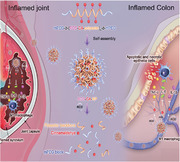

## 1 Introduction

Inflammation is a biological self‐response mechanism to protect the host from injury and infection caused by harmful stimuli.^[^ ^1^ ^]^ However, dysregulated inflammatory responses may drive the development of various inflammatory diseases,^[^ ^2^ ^]^ such as inflammatory bowel disease,^[^ ^3^ ^]^ rheumatoid arthritis (RA),^[^ ^4^ ^]^ and neurodegenerative diseases.^[^ ^5^ ^]^ The general pathogenesis of inflammatory diseases involves the loosening of endothelial junctions, which allows inflammatory cell recruitment and infiltration into inflamed tissues. Subsequently, activated inflammatory cells promote the overproduction of various pro‐inflammatory cytokines, such as tumor necrosis factor‐α (TNF‐α), interleukin‐6 (IL‐6), and interleukin‐1β (IL‐1β), which are responsible for the initiation and progression of inflammatory lesions.^[^ ^2^, ^6^ ^]^ For example, the overproduction of TNF‐α, IL‐6 and IL‐1β in inflamed colon tissues contributes to the onset of ulcerative colitis (UC).^[^ ^7^ ^]^ In RA joints, the high levels of TNF‐α and IL‐1β activate chondrocyte, further leading to cartilage degradation.^[^ ^8^ ^]^ Therefore, the suppression of excessive pro‐inflammatory cytokines is essential for the prevention, alleviation, and treatment of various inflammatory diseases.^[^ ^1^ ^]^

The overproduction of reactive oxygen species (ROS) drives the progression of inflammatory disorders. And the dysregulation of ROS can trigger the generation of pro‐inflammatory cytokines by regulating transcription factors such as nuclear factor‐κB (NF‐κB), which is correlated with the activation of various types of pro‐inflammatory genes expression. Moreover, pro‐inflammatory cytokines, such as TNF‐α and IL‐1β, may amplify the production of ROS, resulting in positive‐feedback loops that accelerate the underlying disease process.^[^ ^9^ ^]^ Therefore, a therapy that carefully controls the enhanced generation of pro‐inflammatory cytokines and ROS is a promising therapeutic option for patients with inflammatory disorders.

Cinnamaldehyde (CA) is a major bioactive component of cinnamon bark oil and has been approved by the Food and Drug Administration as a food additive, indicating its good biosafety.^[^ ^10^ ^]^ CA exhibits anti‐inflammatory and anti‐oxidant activities against inflammatory diseases by regulating inflammatory signaling pathways such as NF‐κB signaling pathway^[^ ^11^ ^]^ and the consumption of free radicals.^[^ ^12^ ^]^ However, the clinical application of CA is limited by its instability due to the rapid oxidation of its aldehyde group, poor solubility, and non‐selectivity for disease sites.^[^ ^10^, ^13^ ^]^ These problems could be resolved by recent advances in smart drug delivery systems, which have opened new avenues for improving drug performance.^[^ ^14^ ^]^

**Figure 1:**
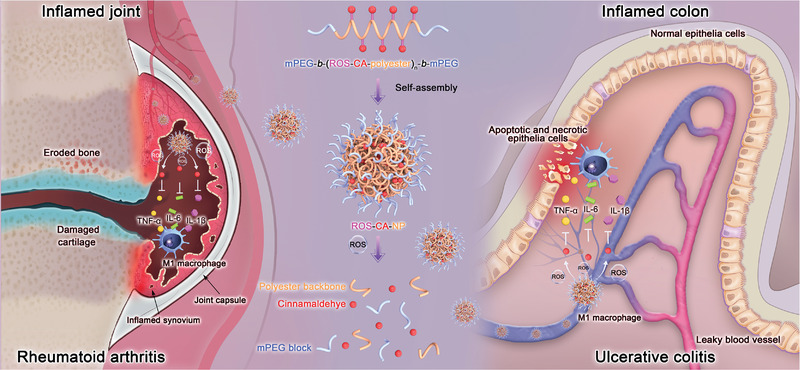
Schematic illustration of the ROS‐responsive polymeric prodrug nanosystem for the treatment of rheumatoid arthritis and ulcerative colitis. The amphiphilic ROS‐responsive polymeric prodrug can self‐assemble into nanoparticles (ROS‐CA‐NPs). After systemic administration, ROS‐CA‐NPs can accumulate at inflamed lesions through leaky blood vessels and release cinnamaldehyde (CA) in response to the oxidative stress to inhibit the overproduction of inflammatory factors in situ, which can attenuate the progression of rheumatoid arthritis and ulcerative colitis. ROS, reactive oxygen species; mPEG, methoxyl polyethylene glycol; TNF‐α, tumor necrosis factor‐α; IL‐6, interleukin‐6; IL‐1β, interleukin‐1β.

In this study, we developed a ROS‐responsive polymeric prodrug nanosystem based on a simple polymer‐drug conjugate (**Figure** **1**). To protect the activity of the functional aldehyde group of CA and achieve controlled drug release, CA was chemically conjugated to the polymer chain via a ROS‐responsive thioacetal linker. This prodrug could self‐assemble into nanoparticles, which could be administered by intravenous injection and accumulate at inflamed sites through leaky vasculature and dysfunctional lymphatic drainage, which are hallmarks of inflammatory pathology.^[^ ^6b^ ^]^ CA was released upon oxidative stress activation. This nanosystem exhibited excellent anti‐inflammatory and ROS‐scavenging activities in vitro. Moreover, animal studies verified that the nanosystem exhibited good biocompatibility and attenuated disease activity. The nanosystem controlled the pathogenesis of inflammation in both mouse models of chronic RA and acute UC. To the best of our knowledge, this is the first time that a ROS‐responsive CA prodrug has been used in the treatment of inflammatory diseases.

## 2 Results and Discussion

### 2.1 Synthesis and Characterization of ROS‐Responsive Polymeric Prodrug Nanoparticles

We initially synthesized a series of amphiphilic ROS‐responsive polymeric prodrugs of CA‐conjugated polyesters with various degrees of polymerization (DPs) via an optimized 1,1,3,3‐tetramethylguanidine‐promoted polyesterification of dibromide and dicarboxylic acid monomers (Scheme S1, Supporting Information).^[^ ^15^ ^]^ Gel permeation chromatography (GPC) results indicated the narrow molecular weight distribution of synthesized products (Table S1, Supporting Information). Proton nuclear magnetic resonance (^1^H NMR) spectroscopy confirmed their structures and DPs (Figures S1–S4, Supporting Information). It was found that the feeding ratio of the dicarboxylic acid monomer and dibromide monomer affected the DPs of the CA‐conjugated polyesters. Three ROS‐responsive CA‐conjugated polymeric prodrugs were obtained: mPEG‐*b*‐(ROS‐CA‐polyester)_2_‐*b*‐mPEG (PP1), mPEG‐*b*‐(ROS‐CA‐polyester)_5_‐*b*‐mPEG (PP2), and mPEG‐*b*‐(ROS‐CA‐polyester)_7_‐*b*‐mPEG (PP3), where the numbers 2, 5, and 7 denote the DPs of the CA‐conjugated polyesters. In addition, a ROS‐nonresponsive CA‐conjugated polymeric prodrug was designed and synthesized, for use as the control (Scheme S2, Supporting Information). The GPC (Table S1, Supporting Information) and ^1^H NMR analyses (Figures S5 and S6, Supporting Information) determined the chemical structures of these ROS‐nonresponsive products and confirmed that they had a narrow molecular weight distribution. The final ROS‐nonresponsive CA‐conjugated polymeric prodrug was denoted as mPEG‐*b*‐(ROS‐CA‐polyester)_4_‐*b*‐mPEG (PP4).

**Figure 2:**
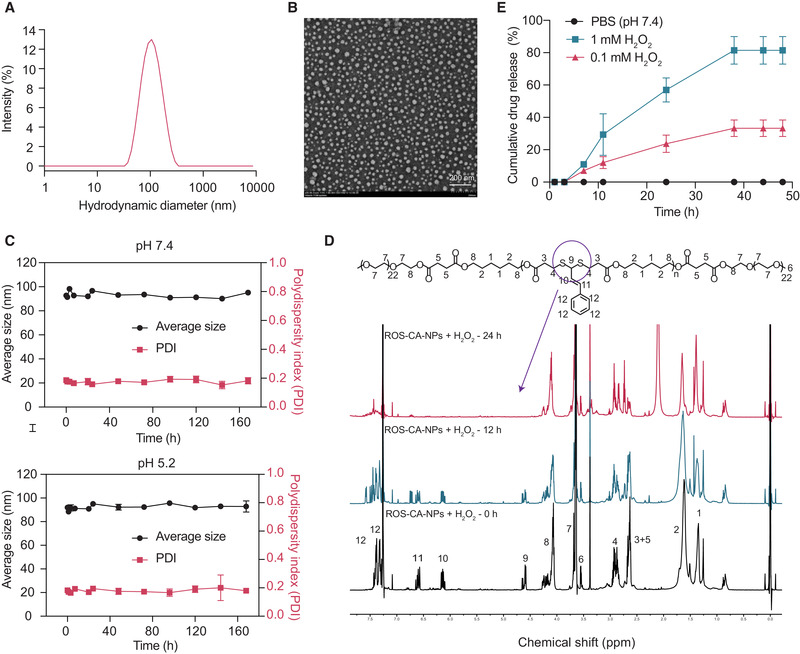
Characterization of ROS‐responsive polymeric prodrug nanoparticles (ROS‐CA‐NPs). A) Average size distribution of ROS‐CA‐NPs. B) Representative transmission electron microscopy image of ROS‐CA‐NPs. C) Stability evaluation of ROS‐CA‐NPs in phosphate‐buffered saline (pH 7.4 or 5.2) at 37 °C for 1 week. D) Proton nuclear magnetic resonance spectra of ROS‐CA‐NPs after incubation with 500 mM hydrogen peroxide (H_2_O_2_) at 37 °C for various periods. E) Cumulative CA release from ROS‐CA‐NPs without or with H_2_O_2_ (0.1 mm and 1 mm) incubation at 37 °C for the stated periods. PDI, polydispersity index.

All nanoparticles were fabricated using a one‐step nanoprecipitation method. To optimize the formulations, the properties of the ROS‐responsive nanoparticles of the CA‐conjugated polyesters with different DPs were investigated by dynamic light scattering (DLS) and transmission electron microscopy (TEM). The results showed that the nanoparticles with two CA‐conjugated units (ROS‐CA_2_‐NPs) had an average hydrodynamic size of 28.8 nm and a high polydispersity index (PDI) value of 0.233 (Figure S7A, Supporting Information). However, no particle morphology was observed in the TEM (Figure S7B, Supporting Information). The nanoparticles with five and seven CA‐conjugated units (ROS‐CA_5_‐NPs and ROS‐CA_7_‐NPs) showed similar average hydrodynamic diameters (95.98 and 91.42 nm, respectively) and small PDI values (0.133 and 0.161, respectively) (**Figure** **2**A; Figure S7C, Supporting Information). In addition, the TEM images showed well‐dispersed nanoparticles (Figure 2B; Figure S7D, Supporting Information). Amphiphilic copolymers of CA‐conjugated polyesters with various DPs lead to the formation of nanoparticles with different stabilities, with a lower DP corresponding with lower stability of nanoparticles. Moreover, the CA drug loading capacity of ROS‐CA_2_‐NPs, ROS‐CA_5_‐NPs and ROS‐CA_7_‐NPs was determined to be 4%, 7.8%, and 9.7%, respectively. Based on the above results, ROS‐CA_2_‐NPs and ROS‐CA_5_‐NPs were excluded from subsequent experiments. ROS‐CA_7_‐NPs are hereinafter referred to as ROS‐CA‐NPs.

The average surface charge of ROS‐CA‐NPs was measured by DLS. These negatively charged nanoparticles had an average zeta potential of ≈−17.4 mV. The result indicates that the nanoparticles have a low tendency to be cleared by the reticuloendothelial system after entering the blood circulation, which can prolong their blood circulation time.^[^ ^16^ ^]^ In addition, the X‐Ray diffraction (XRD) pattern of ROS‐CA‐NPs was further analyzed to characterize the physical existing status (e.g., crystalline state) of nanoparticles.^[^ ^17^ ^,–^ ^19^ ^]^ The ROS‐responsive copolymer (mPEG‐b‐(ROS‐CA‐polyester)_7_‐b‐mPEG), and its nanoparticle form (ROS‐CA‐NPs) were prepared for XRD analysis. As shown in Figure S8 (Supporting Information), there was no difference in terms of the X‐Ray diffraction pattern between copolymer and its nanoparticle form (ROS‐CA‐NPs), indicating that ROS‐CA‐NPs was not in a disordered crystalline‐structure state. Moreover, the critical micelle concentration value of ROS‐CA‐NPs was tested to be 26.3 µg mL^−1^. Next, the stability of ROS‐CA‐NPs under physiological and inflammatory microenvironment conditions was investigated. Over one week at 37 °C in phosphate‐buffered saline (PBS) solution at pH 7.4 or pH 5.2, ROS‐CA‐NPs exhibited only a slight change in their average size and PDI value, indicating ROS‐CA‐NPs had favorable stability (Figure 2C). Subsequently, ROS‐CA‐NPs were incubated with hydrogen peroxide (H_2_O_2_, 500 mM) for various periods and then analyzed by ^1^H NMR spectroscopy to detect any changes in their characteristic peaks (Figure 2D). The gradual decrease of peak 9 suggested that ROS‐sensitive thioacetal linkers were cleaved by ROS as the incubation time increased.

The CA release profiles were further carried out in PBS solution (pH 7.4, with 0.5% Tween 80, 37 °C) with or without the co‐incubation of H_2_O_2_ (Figure 2E). Incubation of ROS‐CA‐NPs without H_2_O_2_ resulted in minimal release of CA. In contrast, an increased release of CA from ROS‐CA‐NPs was observed in the presence of 0.1 mM H_2_O_2_, with ≈30% CA release at 48 h. When the H_2_O_2_ concentration was increased to 1 mM, ≈60% CA was released at 24 h and ≈80% at 48 h. These results demonstrate that ROS can trigger CA release from ROS‐CA‐NPs with a concentration‐dependent behavior

### 2.2 Anti‐Inflammatory Activity and Biocompatibility of ROS‐CA‐NPs in RAW 264.7 Macrophages

**Figure 3:**
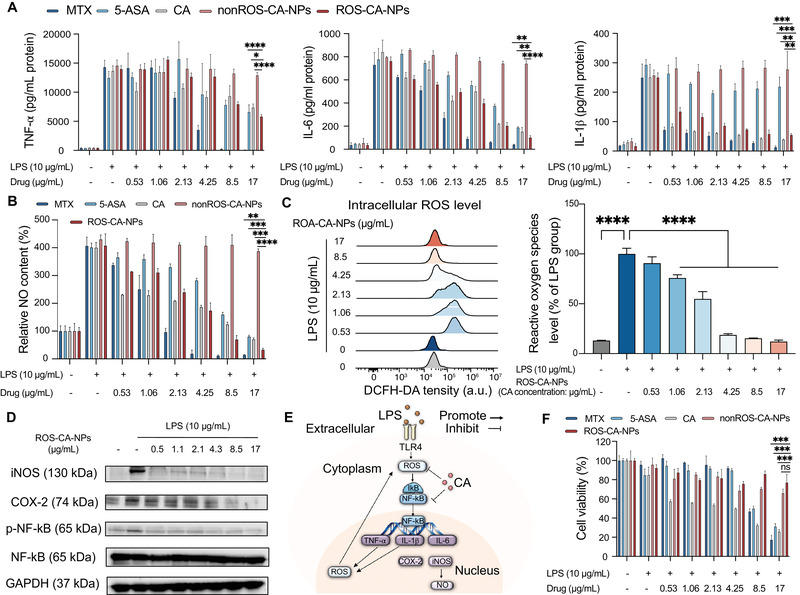
ROS‐responsive polymeric prodrug nanoparticles (ROS‐CA‐NPs) inhibited the LPS‐induced inflammatory responses of RAW 264.7 cells. Cells were incubated with different formulations at various concentrations for 24 h, with or without LPS activation. A) Concentrations of pro‐inflammatory cytokines (TNF‐α, IL‐6, and IL‐1β) were measured using ELISA. Data are presented as means ± SD (*n* = 3). Unpaired Student's t‐test, *p < 0.05, **p < 0.01, ***p < 0.001, and ****p < 0.0001. B) The presence of NO was determined using the Griess reagent. Data are presented as means ± SD (*n* = 3). Unpaired Student's t‐test, **p < 0.01, ***p < 0.001, and ****p < 0.0001. C) Levels of intracellular ROS were qualitatively and quantitatively determined by flow cytometry. Data are presented as means ± SD (*n* = 3). One‐way ANOVA with Tukey's multiple comparisons test, ****p < 0.0001. D) Protein levels of iNOS, COX‐2, NF‐κB, p‐NF‐κB, and GAPDH were determined using western blotting (GAPDH was used as the reference gene). E) The proposed mechanism of anti‐inflammatory and antioxidant effects of CA in LPS‐activated inflammatory responses of RAW 264.7 cells via regulating NF‐κB pathway. F) Cell viability was determined by the MTT assay. Data are presented as means ± SD (*n* = 3). Unpaired Student's t‐test, ns: no significant difference, and ***p < 0.001. ROS‐CA‐NPs or nonROS‐CA‐NPs were added at different CA‐equivalent concentrations. LPS, lipopolysaccharide; 5‐ASA, 5‐aminosalicylic acid; MTX, methotrexate; CA, cinnamaldehyde; TNF‐α, tumor necrosis factor‐α; IL‐6, interleukin‐6; IL‐1β, interleukin‐1β; NO, nitric oxide; a.u., arbitrary units; iNOS, inducible nitric oxide synthase; COX‐2, cyclooxygenase‐2; NF‐κB, nuclear factor‐kappa B; p‐NF‐κB, phosphorylated nuclear factor‐kappa B.

A lipopolysaccharide (LPS)‐induced RAW 264.7 cell model was used in the following in vitro anti‐inflammation studies. Activated macrophages promote the overexpression of pro‐inflammatory cytokines and mediators, which play pivotal roles in the progression of inflammation.^[^ ^20^ ^]^ Therefore, the levels of pro‐inflammatory cytokines (TNF‐α, IL‐6, and IL‐1β) and mediator(nitric oxide (NO)) were examined using the enzyme‐linked immunosorbent assay (ELISA) and the Griess reagent, respectively. Two clinical anti‐inflammatory drugs, methotrexate (MTX) and 5‐aminosalicylic acid (5‐ASA), were used as positive controls. CA‐containing nanoparticles without ROS‐responsive thioacetal linkers (denoted as nonROS‐CA‐NPs), self‐assembled with PP4, were used as a negative control. ELISA results showed that the concentrations of TNF‐α, IL‐6, and IL‐1β in RAW 264.7 cell supernatants increased upon LPS activation, while incubation with ROS‐CA‐NPs dose‐dependently decreased the concentrations of cytokines compared with their concentrations in controls (**Figure** **3**A). In accordance with the ELISA results, excessive NO production resulting from LPS‐mediated activation of RAW 264.7 cells was significantly and dose‐dependently suppressed by ROS‐CA‐NPs treatment. In contrast, the treatment of nonROS‐CA‐NPs failed to inhibit the overproduction of NO, suggesting that CA release was dependent on the presence and activation of the thioacetal linkers. Moreover, free formulations (MTX, 5‐ASA, and CA) showed good inhibition of NO production, but they also showed high cytotoxicity, restricting their clinical applications (Figure 3B).

LPS exposure can promote the overproduction of ROS in the macrophages, and excessive ROS can activate NF‐κB signaling pathway, which further aggravates the progression of inflammation.^[^ ^21^ ^]^ Thus, the levels of ROS and protein levels of the main molecules of NF‐κB signaling pathway in LPS‐stimulated macrophages were measured. Intracellular ROS were stained with 5‐(and‐6)‐chloromethyl‐2′,7′‐dichlorodihydrofluorescein diacetate, which is a general indicator of oxidative stress. The fluorescence signals generated due to the presence of ROS were detected by flow cytometry. Treatment of RAW 264.7 cells with LPS resulted in the formation of high levels of ROS. However, CA‐treated group showed lower concentrations of ROS, indicating an anti‐oxidant effect of free CA. Moreover, compared with nonROS‐CA‐NPs‐treated group, the cellular levels of ROS substantially decreased after incubation with ROS‐CA‐NPs, which demonstrates that ROS‐CA‐NPs dose‐dependently suppressed the production of ROS in LPS‐stimulated macrophages. The result may be ascribed to the cleavage of thioacetal bonds under oxidative stress, causing CA release and subsequently ROS consumption (Figure 3C; Figure S9, Supporting Information). Subsequently, the protein levels of main molecules, such as inducible nitric oxide synthase (iNOS), cyclooxygenase‐2 (COX‐2), NF‐κB, and phosphorylated NF‐κB (p‐NF‐κB) were examined using western blotting. ROS‐CA‐NPs treatment markedly inhibited the LPS‐induced upregulation of iNOS, COX‐2, and p‐NF‐κB protein levels without affecting total NF‐κB expression in a concentration‐dependent manner (Figure 3D). These findings indicate that ROS‐CA‐NPs can consume ROS and suppress NF‐κB pathway to reduce the expression of pro‐inflammatory cytokines and mediators to relieve inflammation in vitro (Figure 3E).

Specifically, the effect of ROS‐CA‐NPs on the viability of with or without LPS‐stimulated RAW 264.7 macrophages was determined using a 3‐(4,5‐dimethylthiazol‐2‐yl)−2,5‐diphenyltetrazolium bromide (MTT) assay. The macrophages showed high viability after incubation with ROS‐CA‐NPs for 24 h at a CA‐equivalent concentration of up to 17 µg mL^−1^ in comparison with controls (Figure 3F; Figure S10, Supporting Information), indicating good biocompatibility of the nanoparticles.

The cellular uptake of the nanoparticles by macrophages of various phenotypes was investigated as well. Compared with M0 phenotype macrophages without LPS pretreatment, the macrophages polarized to the M1 phenotype after LPS activation have stronger phagocytosis.^[^ ^20^ ^]^ Cyanine 5.5 (Cy5.5) dye was physically encapsulated into ROS‐CA‐NPs (Cy5.5‐NPs). Cy5.5‐NPs were then cultured with macrophages with or without LPS activation for 15 min. Compared with M0‐phenotype macrophages, M1‐phenotype macrophages exhibited more efficient uptake of Cy5.5‐NPs, as determined by laser scanning confocal microscopy (LSCM) (Figure S11A, Supporting Information). The cellular uptake behavior was also analyzed by flow cytometry, which also showed that M1‐phenotype macrophages exhibited higher cellular uptake of Cy5.5‐NPs than M0‐phenotype macrophages (Figure S11B, Supporting Information).

### 2.3 Biodistribution of ROS‐CA‐NPs in the Mice with Collagen‐Induced Arthritis

**Figure 4:**
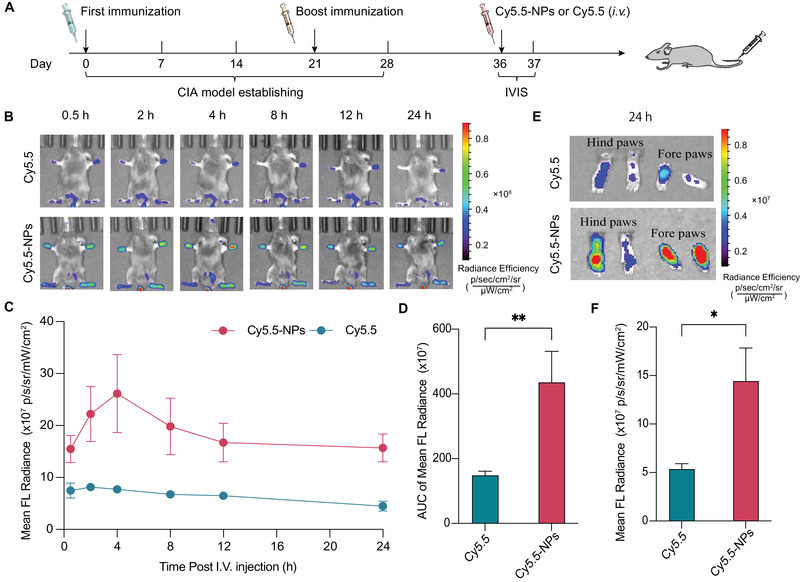
Biodistribution of ROS‐responsive polymeric prodrug nanoparticles (ROS‐CA‐NPs) in the mice with collagen‐induced arthritis (CIA). A) Experimental outline of biodistribution study. B) Time‐dependent in vivo imaging system (IVIS) fluorescence images of the mice with CIA administered with free Cy5.5 or Cy5.5‐NPs. C) Region of interest (ROI) quantification of fluorescence signals in the inflamed paws of the CIA mice over time and D) the area under the curve analysis (AUC). E) Representative images of paws were collected from different groups at the end of the experiment. Fluorescence intensity of Cy5.5 presented in paws of the mice with CIA. F) ROI quantification of fluorescence intensity in dissected paws of the CIA mice at 24 h post‐injection. Data are shown as means ± SD (*n* = 3). Unpaired Student's t test, *p < 0.05, **p < 0.01.

In arthritic joints, the leaky wall of blood vessels results in increased accumulation and prolonged retention of nanoparticles of certain sizes, whereas small molecules are rapidly eliminated from blood circulation. This effect is known as the extravasation through leaky vasculature and subsequent inflammatory cell‐mediated sequestration (ELVIS) effect.^[^ ^22^ ^]^ To investigate the ELVIS effect, the in vivo distribution of free Cy5.5 dye or Cy5.5‐NPs was observed after a single intravenous administration to the mice with collagen‐induced arthritis (CIA) (**Figure** **4**A). Weak fluorescence of Cy5.5 in the inflamed paws of the mice treated with free Cy5.5 was detected after 2 h of systemic administration. This fluorescent signal decayed quickly, suggesting only a small amount of free Cy5.5 accumulated at the arthritic sites. In contrast, Cy5.5‐NPs injected into the mice with CIA showed a strong fluorescent signal in inflamed paws, reaching its highest accumulation at 4 h after administration and then decreasing slowly over 24 h (Figure 4B). The quantification of the region of interest (ROI) against time and the area under the curve in the inflamed paws gave consistent results (Figure 4C,D). These data indicate the preferential accumulation and extended retention of nanoscale materials in arthritic paws due to the ELVIS effect.

To further explore the tissue biodistribution of free Cy5.5 and Cy5.5‐NPs in the mice with CIA, the major organs and paws of the mice were harvested 24 h after injection with free Cy5.5 or Cy5.5‐NPs and ex vivo fluorescence analysis was performed. Although there were considerable fluorescent signals of Cy5.5‐NPs in the liver and kidneys, the concentration of Cy5.5‐NPs was markedly elevated in arthritic paws compared with the concentration of free Cy5.5 (Figure 4E,F; Figure S12, Supporting Information). These results confirmed that Cy5.5‐NPs exhibited arthritic site‐targeting ability, with extended retention time at inflamed sites.

### 2.4 Therapeutic Efficacy of ROS‐CA‐NPs in the Mice with Collagen‐Induced Arthritis

**Figure 5:**
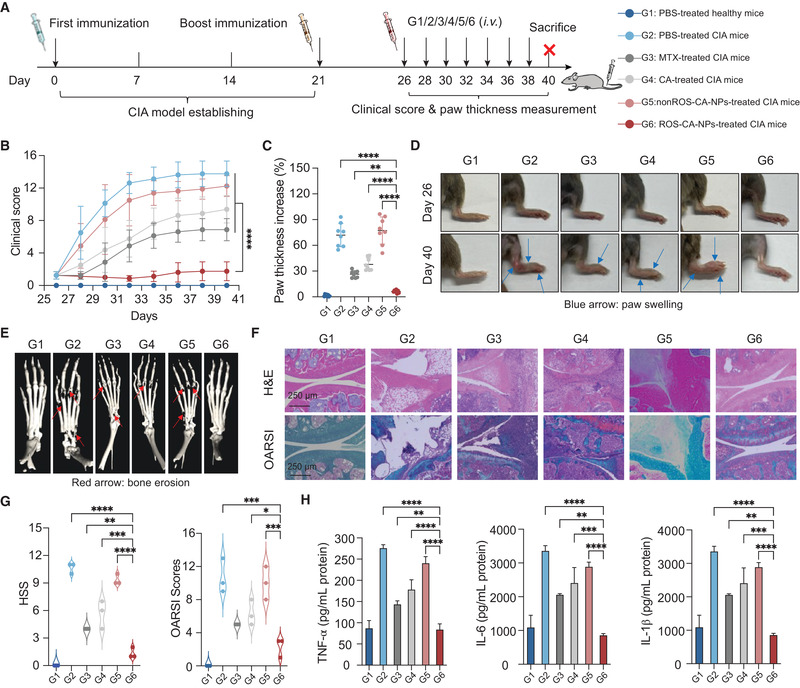
Therapeutic potential of ROS‐responsive polymeric prodrug nanoparticles (ROS‐CA‐NPs) in the mice with collagen‐induced arthritis (CIA). A) Experimental scheme of the therapeutic efficacy study. B) Average clinical scores on different days for the mice in groups G1–G6 during the entire experiment. Data are presented as means ± SD (*n* = 8). Two‐way ANOVA repeated measures with Tukey's multiple comparisons test, ****p < 0.0001. C) Changes in paw thickness in the different treatment groups. Data are presented as means ± SD (*n* = 8). One‐way ANOVA with Tukey's multiple comparisons test, **p < 0.01 and ****p < 0.0001. D) Representative images of hind paws of the mice in each group before (at day 26) and after (at day 40) treatment. E) Representative micro‐computed tomography (CT) images of ankle joints. F) Histological analysis by hematoxylin and eosin (H&E) and safranin‐O staining of ankle joints for each treatment group. Scale bar: 250 µm. G) Histological synovitis score (HSS) and modified Osteoarthritis Research Society International (OARSI) scores based on H&E and safranin‐O staining images. Data are presented as means ± SD (*n* = 3). One‐way ANOVA with Tukey's multiple comparisons test, *p < 0.05, **p < 0.01, ***p < 0.001, and ****p < 0.0001. H) Concentrations of pro‐inflammatory cytokines in paws. Data are presented as means ± SD (*n* = 3). One‐way ANOVA with Tukey's multiple comparisons test, **p < 0.01, ***p < 0.001, and ****p < 0.0001.

Next, we investigated the therapeutic potential of ROS‐CA‐NPs in the mice with CIA, according to the treatment scheme presented in **Figure** **5**A. MTX, the gold standard for the clinical treatment of RA, was used as a positive control. As a comparison, PBS was intravenously injected into eight healthy mice, which were denoted as G1 mice. In addition, forty mice with CIA were randomly divided into five groups (G2–G6), with eight mice per group. These five groups were intravenously administered with a given formulation as follows, G2: PBS; G3: free MTX (5 mg kg^−1^ body weight); G4: free CA (5 mg kg^−1^ body weight); G5: nonROS‐CA‐NPs (CA‐equivalent dose of 5 mg kg^−1^ body weight); and G6: ROS‐CA‐NPs (CA‐equivalent dose of 5 mg kg^−1^ body weight). All groups were administered with the specified formulation seven times every other day. We evaluated the clinical score (Figure 5B; Figure S13, Supporting Information) and measured the paw thickness (Figure 5C) of the mice every other day throughout the study period. Representative images of the hind paws of the mice in the six groups are presented in Figure 5D. The joints of the G1 (PBS‐treated healthy mice) remained in a healthy condition. Compared with the G1 mice, the clinical scores and paw thicknesses were drastically increased and accompanied by severe inflammation in the G2 (PBS) and G5 (nonROS‐CA‐NPs) mice. However, the increase in these signs was slower in the G3 (free MTX) and G4 (free CA) mice. In addition, the G3 mice showed slightly better anti‐inflammatory efficacy than the G4 mice, while the G6 (ROS‐CA‐NPs) mice exhibited the lowest clinical scores and paw thicknesses among all the groups.

To further evaluate the therapeutic efficacy of the formulations, micro‐computed tomography (CT) imaging was used to identify bone erosion in the ankle joints of the mice of the six groups. The reconstructed micro‐CT images (Figure 5E) showed that the G1 (PBS‐treated healthy mice) had smooth bone surfaces, whereas the G2 (PBS) and G5 (nonROS‐CA‐NPs) mice had rough bone surfaces and severe bone erosion in the joints, including the toes and ankles. However, there was only slight bone erosion in the G3 (free MTX) and G4 (free CA) mice. Furthermore, bone erosion was significantly inhibited in the G6 (ROS‐CA‐NPs) mice. At the study endpoint, histological analysis of isolated ankle joints was performed using hematoxylin and eosin (H&E) and safranin‐O staining (Figure 5F). Cartilage destruction, synovial membrane fibrillation, and inflammatory cell infiltration were observed in the G2 (PBS) and G5 (nonROS‐CA‐NPs) mice. Enlargement of the synovial lining cell layer, inflammatory cell infiltration, and pannus formation were observed in the G3 (free MTX) and G4 (free CA) mice. However, the G6 (ROS‐CA‐NPs) mice had structurally sound articular cartilage, and showed dramatically reduced inflammatory cell infiltration and synovial membrane fibrillation, and no pannus formation compared with the G2–G5 mice. Quantitative data were calculated from the images presented in Figure 5F and are presented in Figure 5G. As the overproduction of proinflammatory cytokines contributes to systemic inflammatory progression in RA, the concentrations of three important proinflammatory cytokines (TNF‐α, IL‐1β, and IL‐6) were determined in paws and serum (Figure 5H; Figure S14, Supporting Information). Compared with the G1 (PBS‐treated healthy mice), the concentrations of TNF‐α, IL‐1β, and IL‐6 were increased in the G2 (PBS) and G5 (nonROS‐CA‐NPs) mice. However, the treatment administered to the G3 (free MTX) and G4 (free CA) mice slightly decreased the expression of these three cytokines. In contrast, the G6 (ROS‐CA‐NPs) mice showed the greatest inhibition of the production of these three proinflammatory cytokines. Overall, ROS‐CA‐NPs exhibited promising therapeutic efficacy in a mouse model of RA.

A comprehensive biosafety assessment was also performed for all formulations. H&E staining revealed that there was no tissue damage in the major organs (heart, liver, spleen, lung, and kidneys) of all treatment groups (Figure S15, Supporting Information). The body weight of all mice was monitored every two days, which revealsed no significant changes in body weight throughout the study (Figure S16, Supporting Information). Moreover, the concentrations of key serum biochemical indicators of kidney (blood urea nitrogen and creatinine) and liver (alanine transaminase, aspartate aminotransferase, and alkaline phosphatase) functions were also evaluated (Figure S17, Supporting Information), which revealed that there were no statistically significant between‐group differences in the concentrations of these biochemical indicators. Finally, the hemolytic activities of ROS‐CA‐NPs and nonROS‐CA‐NPs were determined to evaluate their biocompatibility (Figure S18, Supporting Information). The results confirmed their biocompatibility. Thus, all formulations used in this study showed acceptable biosafety.

### 2.5 Biodistribution of ROS‐CA‐NPs in the Mice with Dextran Sulfate Sodium‐Induced Ulcerative Colitis

In the CIA animal study, we have verified the therapeutic efficacy and biosafety of ROS‐CA‐NPs in chronic inflammatory diseases. To further explore the possibility of developing a broad‐spectrum anti‐inflammatory medicine, the anti‐inflammatory efficacy of ROS‐CA‐NPs for the treatment of acute inflammatory diseases was investigated. Dextran sulfate sodium (DSS)‐induced acute UC mouse model was chosen in this study, which displays highly similar pathogenesis to UC patients.^[^ ^23^ ^]^

**Figure 6:**
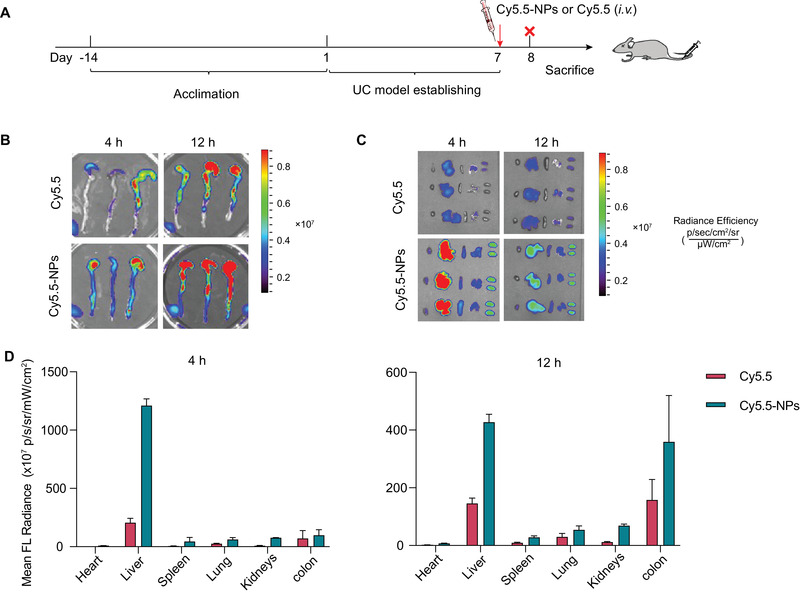
Biodistribution of ROS‐responsive polymeric prodrug nanoparticles (ROS‐CA‐NPs) in the mice with ulcerative colitis (UC). A) Experimental outline of biodistribution study. B) Ex vivo fluorescence images of colons were collected from the mice with UC at 4 and 12 h after the injection of free Cy5.5 or Cy 5.5‐NPs. C) Ex vivo fluorescence images of major organs (heart, liver, spleen, lung, and kidneys) were collected from the mice with UC at 4 h and 12 h after the injection of free Cy5.5 or Cy 5.5‐NPs. D) ROI quantification of fluorescence intensity in dissected major organs and colons of the mice with UC at 4 and 12 h post‐injection of free Cy5.5 or Cy5.5‐NPs.

Nanodrugs of certain sizes (10 to 200 nm) may passively aggregate at an inflammatory site via the defective intestinal barrier that occurs at disease sites. This process is known as the enhanced epithelial permeability and retention effect.^[^ ^24^ ^]^ To determine whether a similar effect occurred with our system, the biodistribution of free Cy5.5 and Cy5.5‐NPs were studied in the mice with UC after a single intravenous injection (**Figure** **6**A). After the injection of free Cy5.5, weak fluorescent signals were observed in the diseased colons of the mice with UC. In contrast, after the injection of Cy5.5‐NPs, the mice with UC had strong fluorescent signals in their inflamed colons, as desired (Figure 6B). Moreover, there were relatively strong fluorescent signals in the kidneys and liver, demonstrating that intravenously injected nanoparticles were mainly eliminated by renal or hepatic clearance (Figure 6C). ROI quantification of fluorescence intensity in dissected major organs and colons at 4 and 12 h post‐injection of free Cy5.5 or Cy5.5‐NPs gave consistent results (Figure 6D).

### 2.6 Therapeutic Efficacy of ROS‐CA‐NPs in the Mice with Dextran Sulfate Sodium‐Induced Ulcerative Colitis

**Figure 7:**
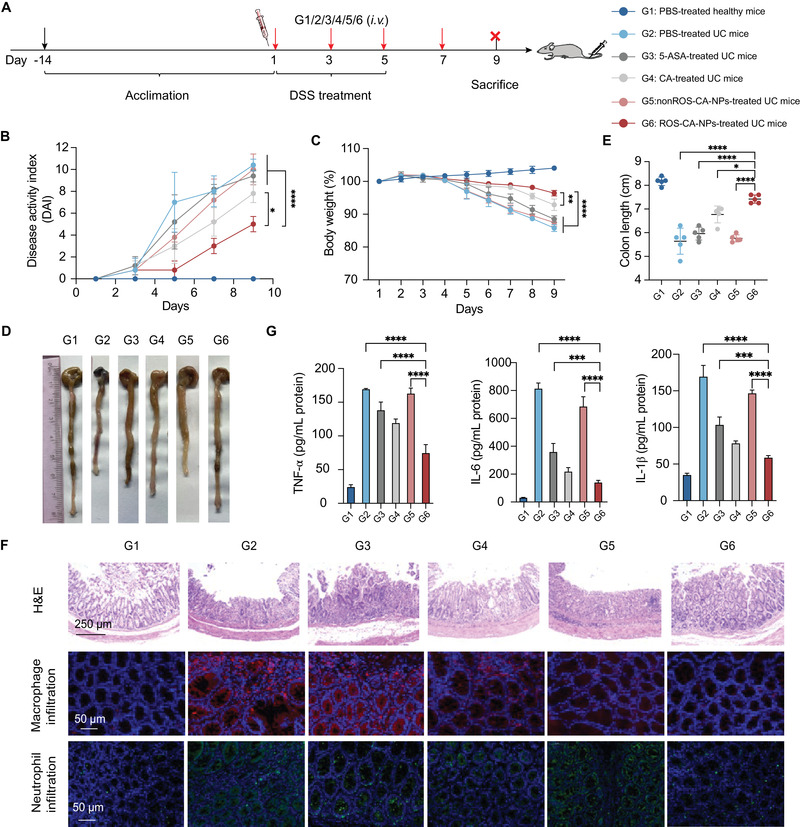
Therapeutic potential of ROS‐responsive polymeric prodrug nanoparticles (ROS‐CA‐NPs) in the mice with ulcerative colitis (UC). A) Experimental scheme of the therapeutic efficacy study. B) Disease activity index scores (DAI) of G1–G6 mice during the entire treatment period. Data are presented as means ± SD (*n* = 5). Two‐way ANOVA repeated measures with Tukey's multiple comparisons test, *p < 0.05 and ****p < 0.0001. C) Body weight changes with time for G1–G6 mice during the entire treatment period. Data are presented as means ± SD (*n* = 5). Two‐way ANOVA repeated measures with Tukey's multiple comparisons test, **p < 0.01 and ****p < 0.0001. D) Representative images of colons extracted from G1–G6 mice at the end of the treatment period. E) Colon length of G1–G6 mice at the end of the treatment period. Data are presented as means ± SD (*n* = 5). One‐way ANOVA with Tukey's multiple comparisons test, *p < 0.05 and ****p < 0.0001. F) Hematoxylin and eosin (H&E) stained images of colonic tissues and immunofluorescence staining of colonic tissues indicated macrophage infiltration (red signal) or neutrophil infiltration (green signal). Blue signal represents nuclear staining with 4′,6‐diamidino‐2‐phenylindole. G) Concentrations of pro‐inflammatory cytokines in serum. Data are presented as means ± SD (*n* = 3). One‐way ANOVA with Tukey's multiple comparisons test, ****p < 0.001 and ****p < 0.0001.

The therapeutic efficacy of ROS‐CA‐NPs was further evaluated in the mice with UC. 5‐ASA, a clinical anti‐inflammatory drug used for the treatment of UC, was selected as a positive control. As a comparison, PBS was intravenously injected into five healthy mice, which were denoted as G1 mice. In addition, twenty‐five mice with UC were randomly divided into five groups (G2–G6), with five mice per group. These five groups were intravenously administered with a given formulation as follows, G2: PBS; G3: free 5‐ASA (10 mg kg^−1^ body weight); G4: free CA (10 mg kg^−1^ body weight); G5: nonROS‐CA‐NPs (CA‐equivalent dose of 10 mg kg^−1^ body weight); and G6: ROS‐CA‐NPs (CA‐equivalent dose of 10 mg kg^−1^ body weight). All groups were administered with the specified formulation four times every other day. **Figure** **7**A presents the overall experimental design for UC treatment. Therapeutic efficacy was assessed using various parameters: the disease activity index (DAI) score, colon length, the expression levels of pro‐inflammatory cytokines, the colon histology, and the amount of inflammatory cell infiltration. During the entire experimental period, the DAI score, which quantifies body‐weight change, stool consistency, and rectal bleeding,^[^ ^25^ ^]^ was recorded every other day for each mouse. As shown in Figure 7B,C, there was no difference in the DAI scores or body weight of G1 (PBS‐treated healthy mice) before and after treatment. However, G2–G5 mice had increased DAI scores and decreased body weight, together with watery diarrhea and bleeding, consistent with the presence of severe colitis. In contrast, G6 (ROS‐CA‐NPs) mice had enhanced stool consistency, reduced visible fecal bleeding, and non‐severe changes in body weight. As colon shortening is a key parameter for the assessment of the severity of colitis,^[^ ^26^ ^]^ at the end of the treatment period, the mice were sacrificed. Their colons were isolated and imaged (Figure 7D) and the length of the colons was measured (Figure 7E). The colons of G2–G5 mice with UC were shorter than those of healthy mice (G1). However, G6 (ROS‐CA‐NPs) mice had significantly less colon shortening.

Next, the degree of architectural integrity and the infiltration of inflammatory cells into colonic tissues was evaluated by histological analysis of H&E‐stained tissues. Colon sections of G1 (PBS‐treated healthy mice) exhibited perfect colonic morphology, with no inflammatory cell infiltration. In contrast, G2 (PBS) and G5 (nonROS‐CA‐NPs) mice showed a loss of intact regular colonic morphology, an increase in submucosal layer thickness, and an excess of inflammatory cells infiltrating the mucosa. Moreover, G3 (free 5‐ASA) and G4 (free CA) mice showed comparatively intact colonic architecture but displayed inflammatory cell infiltration and thickening of the submucosal layer. In comparison, G6 (ROS‐CA‐NPs) mice retained the integrity of their colonic structure and showed reduced inflammatory cell infiltration compared with G2–G5 mice. Subsequently, the infiltration of inflammatory cells, such as macrophages and neutrophils, into the inflamed colons was specifically investigated. Weak fluorescent signals were observed in G1 mice, revealing the low presence of neutrophils and macrophages in healthy colons. However, a significant increase in neutrophil and macrophage infiltration was seen in G2 (PBS) and G5 (nonROS‐CA‐NPs) mice, indicating the presence of severe UC. Considerable infiltration was also present in G3 (free 5‐ASA) and G4 (free CA) mice, demonstrating that the free drugs partially reduced neutrophil and macrophage infiltration into colonic tissues. A more efficient reduction of inflammatory cell infiltration was observed in G6 (ROS‐CA‐NPs) mice, revealing a decrease in the severity of UC (Figure 7F). Blood samples were also collected from the mice at the end of the treatment period. The concentrations of pro‐inflammatory cytokines (TNF‐α, IL‐1β, and IL‐6) in serum were determined. The G6 group showed lower concentration of pro‐inflammatory cytokines than the G2–G5 groups, revealing the excellent anti‐inflammatory effect of ROS‐CA‐NPs in the mice with UC (Figure 7G).

Finally, the biosafety of the therapeutic agents was evaluated in vivo. Major organs were stained with H&E for histological analysis. Serum was isolated from whole‐blood samples for biochemical analysis. Tissue damage was not observed in any of the treatment groups (Figure S19, Supporting Information). We further evaluated important indicators of kidney (blood urea nitrogen and creatinine) and liver (alanine transaminase and aspartate aminotransferase) functions in serum samples from all mice (Figure S20, Supporting Information). The results showed no statistical between‐group differences in the concentrations of these indicators. Therefore, all of the formulations used in this study showed the desired level of biosafety.

## 3 Conclusion

Inflammation is closely related to the etiology and pathogenesis of various acute and chronic inflammatory diseases, such as UC and RA. Anti‐inflammatory agents are a promising approach for inflammatory disease management. However, the application of currently used anti‐inflammatory drugs remains restricted by insufficient efficacy and the fact that they can increase the risk of severe infections and malignancies.^[^ ^27^ ^]^ Given the limitations of currently used anti‐inflammatory drugs, drug delivery systems are needed to reduce off‐target side effects and improve therapeutic efficacy. To precisely control drug biodistribution and release, inflammation‐responsive drug‐delivery systems have been widely investigated.^[^ ^28^ ^]^

Considering the overproduction of ROS at inflamed sites, ROS‐responsive drug delivery systems have been extensively explored in various animal models of acute and chronic inflammatory diseases. The rational design of ROS‐sensitive systems enables on‐demand drug release at inflamed sites, with improved therapeutic efficacy in vitro and in vivo, indicating that these systems are a promising anti‐inflammatory approach.

However, some problems remain to be solved to develop ROS‐responsive drug delivery systems for the treatment of RA and UC. Currently, anti‐inflammatory drugs are associated with carriers via hydrophobic interactions, hydrogen bonding, π–π stacking, or electrostatic interactions. These are weak physical bonds and thus may lead to premature drug release in the blood circulation, i.e., drug release before the systems reach their designated sites, resulting in adverse effects on healthy tissues.^[^ ^29^ ^]^ Moreover, prematurely released drugs may react with substances in the body, resulting in decrease in drugs’ pharmacological activity and therapeutic efficacy. To avoid such problems, a prodrug strategy is employed, whereby drugs are directly bonded to drug carriers to reduce premature drug release and protect drug activity.^[^ ^30^ ^]^

CA was selected as the anti‐inflammatory drug in this study, as the therapeutic efficacy of free CA has been confirmed in many animal models of inflammatory diseases, such as RA^[^ ^11^, ^12^ ^]^ and UC.^[^ ^31^ ^]^ Moreover, unlike other clinical anti‐inflammatory drugs, CA possesses an aldehyde functional group, which can react with a thiol linker to form a thioacetal bond, which provides an opportunity for CA to form ROS‐responsive prodrugs. Unlike other ROS‐sensitive groups, thioacetal linkers can be easily obtained through a one‐step reaction of aldehyde and thiol groups, and they exhibit good resistance to enzyme‐, acid‐ and base‐mediated degradation.^[^ ^1^, ^28^ ^]^ The ROS responsiveness of thioacetal bonds has been verified in previous studies of cancer therapeutics,^[^ ^32^ ^]^ which indicates the feasibility of their use at inflammatory sites that produce high levels of ROS.

In this study, a ROS‐responsive polymeric prodrug nanosystem was developed for the treatment of RA and UC. A series of CA‐linked amphiphilic polymeric prodrugs with different CA‐conjugated units were synthesized. The most effective polymeric prodrug was determined based on size, morphology, and drug‐loading capacity. In vitro studies demonstrated that the drug released from the prodrug had significant inhibitory effects on inflammatory mediators and ROS levels, together with low cytotoxicity. To investigate the therapeutic efficacy of the system in chronic inflammatory diseases, a mouse model of RA was constructed using collagen. A biodistribution study confirmed that compared with free small molecules, ROS‐CA‐NPs accumulated more and were retained for a longer time in inflamed joints. In vivo therapeutic studies indicated that the ROS‐CA‐NPs showed improved therapeutic efficacy and reduced side effects in inflamed tissues compared with the free drugs. No organ damage was caused by any of the formulations, indicating the excellent biosafety of ROS‐CA‐NPs for treating chronic inflammatory disease. To further evaluate the anti‐inflammatory efficacy of ROS‐CA‐NPs in acute inflammatory diseases, a mouse model of UC was developed by oral administration of 3% (w/v) dextran sulfate sodium salt. In vivo fluorescence imaging indicated that ROS‐CA‐NPs exhibited passive targeting and a longer retention time in inflamed colons compared with free small molecules. Moreover, therapeutic studies showed that ROS‐CA‐NPs attenuated clinical disease activities and inhibited pro‐inflammatory cell infiltration in inflamed colons. Finally, none of the formulations caused organ toxicity.

The findings of this study indicate the feasibility of using a simple ROS‐responsive polymeric prodrug nanosystem to achieve on‐demand drug release at inflamed sites. The system demonstrated greater therapeutic efficacy than traditional clinical drugs for the treatment of RA and UC. This system has the potential to be developed for clinical trials as an effective and safe nanotherapeutic strategy for inflammatory diseases.

## 4 Experimental Section

### Materials

Trans‐cinnamaldehyde (CA, Tokyo Chemical Industry), 1,1,3,3‐tetramethylguanidine (TMG, Tokyo Chemical Industry), and 1,6‐dibromohexane (Dieckmann) were purchased from Dieckmann (Shenzhen, China); 3‐mercaptopropionic acid (3‐MPA) was purchased from Macklin (Shanghai, China); dextran sulfate sodium (DSS) salt was purchased from Meilun Biotech Co., Ltd. (Dalian, China); methoxy (polyethylene glycol)1000‐hydroxyl was purchased from Ponsure (Shanghai, China); LPS (Sigma–Aldrich) and modified Griess reagent (Sigma–Aldrich) were purchased from Tin Hang Technology Limited (Hong Kong, China); 3‐(4,5‐dimethylthiazol‐2‐yl)−2,5‐diphenyltetrazolium bromide (MTT) was purchased from J&K Scientific (Hong Kong, China); the CM‐H2DCFDA ROS probe, 4% (w/w) paraformaldehyde, 4′,6‐diamidino‐2‐phenylindole, and enzyme‐linked immunosorbent assay (ELISA) kits were purchased from ThermoFisher Scientific (Hong Kong, China); bovine type‐II collagen (Chondrex), complete Freund's adjuvant (Chondrex) and incomplete Freund's adjuvant (Chondrex) were purchased from Biolead (Beijing, China); and the custom LEGENDplex^TM^ mouse inflammation panel (Biolegend) was purchased from Dakewe (Beijing, China).

### Synthesis of ROS‐Responsive CA‐Conjugated Monomers

The ROS‐responsive CA‐conjugated monomer was synthesized according to a previously reported method.^[^ ^32b^ ^]^ Briefly, CA (100 mmol, 13.2 g) and 3‐MPA (240 mmol, 25 g) were completely dissolved in 80 mL of ethyl acetate. A few drops of trifluoroacetic acid were added to the mixture in an ice‐water bath, protected from light. After 24 h of reaction, the crude product was purified by cold water and hexane, alternately, three times. The final product was obtained after drying in a vacuum oven overnight to obtain a white solid, namely, an ROS‐CA‐monomer.

### Synthesis of Bromide‐Terminated ROS‐Responsive CA‐Conjugated Polyesters

Bromide‐terminated polyesters were synthesized via TMG‐promoted polyesterification. In general, the ROS‐CA‐monomer and 1,6‐dibromohexane were dissolved in dimethyl sulfoxide (DMSO), and TMG was then added to the mixture. The initial molar ratio of the ROS‐CA‐monomer, 1,6‐dibromohexane, and TMG was m:1:2 × m. After 16 h of reaction at 40 °C, the product was precipitated in a large amount of deionized water and collected after drying in a vacuum oven overnight. The resulting product was bromide‐terminated (ROS‐CA‐polyester)_n_ (m<1, different m contributed to different n).

### Synthesis of ROS‐Responsive CA‐Conjugated Polymeric Prodrugs

Bromide‐terminated (ROS‐CA‐polyester)_n_ was dissolved in DMSO, and a mixture of mPEG1000‐COOH and TMG was then introduced into the above solution. The initial molar ratio of (ROS‐CA‐polyester)_n_, mPEG1000‐COOH, and TMG was 1:1.2:1.2. The solution was stirred for 8 h at 40°C and the resulting product was then purified by dialysis against deionized water for two days in the dark to obtain mPEG‐*b*‐(ROS‐CA‐polyester)_n_‐*b*‐mPEG.

### Preparation and Characterization of CA‐Conjugated Nanoparticles

ROS‐CA‐NPs and nonROS‐CA‐NPs were formed using a modified nanoprecipitation technique.^[^ ^33^ ^]^ Briefly, 1 mL of tetrahydrofuran (THF) solution containing 20 mg of mPEG‐*b*‐(ROS‐CA‐polyester)_n_‐*b*‐mPEG or mPEG‐*b*‐(nonROS‐CA‐polyester)_n_‐*b*‐mPEG amphiphilic copolymers was quickly pipetted into 4 mL of deionized water under a strong vortex. The solution was then dialyzed against deionized water for one day to remove the THF.

### ROS Responsiveness

^1^H NMR spectra were determined to investigate the structural change in ROS‐CA‐NPs after incubation with an ROS reagent (H_2_O_2_). Briefly, 0.5 mL of nanoparticle solution (1.23 mg) was treated with 0.5 mL of H_2_O_2_ (1 m) at 37 °C for 0, 12, and 24 h. The solution was then freeze‐dried and the resulting powder was dissolved in chloroform‐d (CDCl_3_) for ^1^H NMR analysis.

### ROS‐Triggered Drug Release

The CA release profile of ROS‐CA‐NPs with or without H_2_O_2_ (0.1 mm, 1 mm) at 37 °C was determined by dialysis method. 1 mL of ROS‐CA‐NPs was dialysis in a 3500 Da‐cutoff dialysis bag against 9 mL of PBS (pH 7.4, with 0.5% Tween 80) with or without H_2_O_2_ (0.1 mm, 1 mm). The outer solution was completely replaced at each time and fresh PBS was subsequently added. The cumulative release percentage of CA over time was calculated by HPLC measurement.

### Cell Culture

Mouse macrophage‐like cells (RAW 264.7 cells, from the American Type Culture Collection) were purchased from Shanghai Guandao Bio‐Chem Technology Co., Ltd. (Shanghai, China). In general, RAW 264.7 cells were cultured in Dulbecco's modified Eagle's medium (DMEM) at 37°C under 5% carbon dioxide. The medium was prepared with 1% (v/v) penicillin/streptomycin and 10% (v/v) fetal bovine serum (FBS). The cells were subcultured every other day.

### ELISA

RAW 264.7 cells were seeded in 24‐well plates and cultured for 24 h. The medium was then removed and replaced with fresh medium. Formulations (free MTX, free 5‐ASA, free CA, nonROS‐CA‐NPs and ROS‐CA‐NPs) at various concentrations were introduced into each well and the cells were incubated for 2 h, after which they were co‐cultured with or without LPS (10 µg per well) for another 22 h. ROS‐CA‐NPs or nonROS‐CA‐NPs were added at different CA‐equivalent concentrations. To determine the concentrations of pro‐inflammatory cytokines (TNF‐α, IL‐6, and IL‐1β) in vitro, the cell culture supernatants were collected and analyzed immediately using ELISA kits, according to the manufacturer's instructions. The absorbance of the samples was measured at 450 nm using a multi‐mode microplate reader.

### Determination of Nitric Oxide Concentration

The concentration of nitric oxide in the cell culture supernatants of stimulated RAW 264.7 cells was determined using Griess reagent. RAW 264.7 cells were treated with formulations (free MTX, free 5‐ASA, free CA, nonROS‐CA‐NPs and ROS‐CA‐NPs) at different concentrations using the same procedure described in the section of ELISA. ROS‐CA‐NPs or nonROS‐CA‐NPs were added at different CA‐equivalent concentrations. The cell culture supernatant was then reacted with an equal volume of freshly prepared Griess reagent (40 mg mL^−1^ in deionized water) at room temperature in the dark for 15 min. Finally, the absorbance was measured at 540 nm using a multi‐mode microplate reader.

### ROS Determination

RAW 264.7 cells were treated with free CA or nonROS‐CA‐NPs or ROS‐CA‐NPs at different CA‐equivalent concentrations using the same procedure described in the section of ELISA. The cells were washed with PBS and stained with 300 µL of FBS‐free DMEM containing CM‐H2DCFDA (10 µM) at 37 °C for 20 min. The cells were then washed and suspended in PBS. Finally, fluorescence signals were detected by flow cytometry using the fluorescein isothiocyanate channel. FlowJo software (BD Biosciences, Franklin Lakes, NJ, USA) was used to analyze the data.

### Western Blotting Assay

The protein expression levels of the main molecules of NF‐κB signaling pathway in LPS‐stimulated macrophages were examined using western blotting. RAW 264.7 cells were treated with ROS‐CA‐NPs at different CA‐equivalent concentrations using the same procedure described in the section of ELISA. Total cellular protein was extracted on ice using a radioimmunoprecipitation assay buffer with loading buffer and a phosphatase inhibitor cocktail. The cell lysates were separated by 10% sodium dodecyl sulfate–polyacrylamide gel electrophoresis and then transferred onto nitrocellulose membranes. The membranes were blocked with bovine serum albumin for 1 h and then probed with specific primary antibodies against the main molecules of NF‐κB signaling pathway and GAPDH, separately, at 4 °C overnight. Subsequently, the membranes were washed with buffer and incubated with secondary antibodies at room temperature for 1 h. Finally, the protein blots were visualized using an enhanced chemiluminescence kit and imaged with a ChemiDoc Imaging System (Bio‐Rad, Hercules, CA, USA).

### Cell Viability Assay

Cell viability was determined using an MTT assay. RAW 264.7 cells were treated with formulations (free MTX, free 5‐ASA, free CA, nonROS‐CA‐NPs, and ROS‐CA‐NPs) at different concentrations using the same procedure described in the section of ELISA. ROS‐CA‐NPs or nonROS‐CA‐NPs were added at different CA‐equivalent concentrations. An MTT solution (50 µL, 5 mg mL^−1^) was then added to each well and the cells were cultured for a further 3 h. The MTT‐containing medium was then removed by vacuum and DMSO was added to dissolve the formazan crystals. Finally, the absorbance was measured at 570 and 630 nm using a multi‐mode microplate reader to calculate cell viability.

### Animals

Male DBA/1 mice aged 6–8 weeks for the RA study and female C57BL/6 mice aged 8–10 weeks for the UC study were purchased from Beijing Vital River Company (Beijing, China). Ethical approval for the animal studies was obtained from the Institutional Animal Care and Use Committee of The Second Affiliated Hospital of Guangzhou MedicalUniversity. Animal welfare was guaranteed during all animal studies.

### CIA Model

A mouse model of CIA was developed by double immunization. Male DBA/1 mice were intradermally injected with bovine type‐II collagen (2 mg mL^−1^) emulsified in an equal volume of complete Freund's adjuvant (4 mg mL^−1^) in their back, near the tail. After 21 days to allow fully developed immunity, a booster immunization emulsion of bovine type‐II collagen (2 mg mL^−1^) and incomplete Freund's adjuvant (4 mg mL^−1^) was given to the mice.

### Biodistribution Study in CIA Mice

Previous studies had reported that nanoparticles and macromolecules of certain sizes tend to passively accumulate in inflamed joints via the ELVIS effect. To verify this effect, free Cy5.5 and Cy5.5‐loaded nanoparticles (Cy5.5‐NPs) were intravenously administrated to CIA mice with advanced arthritis (clinical score ranged from 10 to 12). The fluorescent signal of Cy5.5 in paws was monitored at 0.5, 2, 4, 8, 12, and 24 h post‐injection at an excitation wavelength of 675 nm using an in vivo imaging system. At the end of test, the CIA mice were sacrificed, and the major organs (including heart, liver, spleen, lung, and kidneys) and paws were collected for ex vivo imaging analysis via Cy5.5 fluorescent signals.

### Therapeutic Efficacy and Biosafety Assessment in CIA Mice

Mice with early‐stage CIA (clinical score ranged from 1 to 2) were selected for therapeutic efficacy studies. CIA mice were divided into five groups (*n* = 8), and intravenously administrated PBS, free MTX (5 mg kg^−1^ per body weight), free CA (5 mg kg^−1^ per body weight), nonROS‐CA‐NPs (CA‐equivalent concentration: 5 mg kg^−1^ per body weight), or ROS‐CA‐NPs (CA‐equivalent concentration: 5 mg kg^−1^ per body weight) via the tail vein every other day. Eight healthy DBA/1 mice served as a control group and were intravenously injected with PBS.

The severity of arthritis was evaluated every other day following previously published protocols.^[^ ^6^, ^34^ ^]^ The clinical scores were summed for each mouse, with a maximum possible score of 16. The paw thickness and body weight of each mouse were recorded every two days. After 14 days of treatment, the mice were sacrificed, and blood was collected for the quantification of serum proinflammatory cytokines and key biochemical markers. The major organs (heart, liver, spleen, lung, and kidneys) were harvested for H&E staining. Ankle joints were isolated for H&E and safranin O staining. Histological synovitis and Osteoarthritis Research Society International scores were calculated to evaluate the arthritic status of the joints.^[^ ^35^ ^]^ Paws were collected for proinflammatory cytokine detection and bone erosion assessment via microcomputed tomography (micro‐CT) imaging analysis. The micro‐CT imaging parameters were as follows: voltage, 65 kV; current, 185 µA; field of view, 59.18 mm × 59.18 mm; and spot size, 50 µm.

### UC Model

A mouse model of acute colitis was induced by adding DSS (MW 36000–50000) to the drinking water. In detail, after two weeks of acclimation, female C57BL/6 mice were given 3% DSS (w/v) in their drinking water, starting from day one, for five continuous days. The DSS solution was replaced with a fresh solution every other day. On day six, the DSS‐containing drinking water was replaced with regular drinking water for an additional four days.

### Biodistribution Study in UC Mice

To evaluate the passive targeting ability of nanoscale materials in the inflamed colon, free Cy5.5 and Cy5.5‐NPs were intravenously administrated to UC mice. Four or twelve hours after injection, the mice were sacrificed, and the major organs (heart, liver, spleen, lung, kidneys, and colon) were collected. Fluorescence images of the major organs were captured at an excitation wavelength of 675 nm using an ex vivo imaging system.

### Therapeutic Efficacy and Biosafety Assessment in UC Mice

After two weeks of acclimation, 30 female C57BL/6 mice were randomly divided into six groups (n = 5 per group). Acute UC was induced in five of the groups by adding 3% DSS (w/v) to the drinking water. These five groups of UC mice were then treated with PBS, free 5‐ASA (10 mg kg^−1^ per body weight), free CA (10 mg kg^−1^ per body weight), nonROS‐CA‐NPs (CA‐equivalent concentration: 10 mg kg^−1^ per body weight), or ROS‐CA‐NPs (CA‐equivalent concentration: 10 mg kg^−1^ per body weight) by tail vein administration every two days for a total of four injections. Healthy mice served as a control group and received an equal volume of PBS.

During the entire study, the DAI, a clinical measure combining the scores of three symptoms‐body weight loss, stool consistency, and rectal bleeding‐was calculated every other day using a standard scoring system.^[^ ^25^ ^]^ At the end of the treatment period (day nine), all mice were sacrificed and blood was collected for biochemical and pro‐inflammatory cytokine evaluations. The major organs (heart, liver, spleen, lung, and kidneys) were harvested and stained with H&E for histological analysis. Meanwhile, the colon was isolated and imaged. The length of the colon was measured using a ruler and, subsequently, the colon was sectioned for H&E, anti‐MPO, and anti‐F4/80 staining.

### Statistical Analysis

All statistical tests were performed using Prism version 9.0 software (GraphPad Software Inc., San Diego, CA, USA). Student's t‐test and one‐way ANOVA were applied for analysis comparing two samples and multiple samples, respectively. To investigate the effect of two parameters and their interaction, two‐way ANOVA was used. Data were presented as the means ± SD. In general, *p* < 0.05 was considered to be statistically significant.

## Conflict of Interest

A US provisional patent application was filed with No. 63/515,837.

## Author Contributions

Y.Z. and L.L. contributed equally to this work. Y.Z., Z.L., and W.W. created this project. Y.Z., L.L., T.W., C.M. and P.S. performed the experimental work. Y.Z. and L.L analyzed the data. Y.Z., C.L., Z.L, W.G. and W.W. wrote the manuscript. C.L., Z.L., W.G. and W.W. supervised the project. All authors discussed the results and contributed to the final manuscript.

## Supporting information

Supporting Information

## Data Availability

The data that support the findings of this study are available from the corresponding author upon reasonable request.
