# Supplementary material for: Reactive Oxygen Species‐Responsive Polymeric Prodrug Nanoparticles for Selective and Effective Treatment of Inflammatory Diseases
Source: Adv Healthc Mater. 2023 Aug 17;12(29):2301394. doi: 10.1002/adhm.202301394 (PMC11468797; doi:10.1002/adhm.202301394)
Supplement: Supplementary file 1 — Supporting Information [file ADHM-12-2301394-s001.pdf]

ADVANCED HEALTHCARE MATERIALS

Supporting Information

for *Adv. Healthcare Mater.*, DOI 10.1002/adhm.202301394

Reactive Oxygen Species-Responsive Polymeric Prodrug Nanoparticles for Selective and Effective Treatment of Inflammatory Diseases

Yaming Zhang, Lu Liu, Tianyi Wang, Cong Mao, Pengfei Shan, Chak Sing Lau, Zhongyu Li, Weisheng Guo* and Weiping Wang**

Supporting Information

Reactive oxygen species-responsive polymeric prodrug nanoparticles for selective and effective treatment of inflammatory diseases

Yaming Zhang†, Lu Liu†, Tianyi Wang, Cong Mao, Pengfei Shan, Chak Sing Lau, Zhongyu Li, Weisheng Guo*, Weiping Wang**

† These authors contributed equally to this work.

*Corresponding author. Email: lizy@wzu.edu.cn (Z.L.) tjuguoweisheng@126.com (W.G.) wangwp@hku.hk (W.W.)

Materials: Trifluoroacetic acid (TFA, Sigma-Aldrich) was purchased from Tin Hang Technology Limited (Hong Kong, China). Ditert-butyl (2S)-2-aminobutanedioate, (E)-3-phenylprop-2-enoic acid, anhydrous 1-hydroxybenzotriazole (HOBt), 1-(3-dimethylaminopropyl)-3-ethylcarbodiimide (EDCI), N, N-ethyldiisopropylamine (DIEA), anhydrous sodium sulfate (Na_2SO_4) and succinic anhydride were purchased from Macklin (Shanghai, China). Cyanine 5.5 NHS ester (Cy5.5, Lumiprobe) was purchased from Xi'an ruixi Biological Technology Co., Ltd. (Xi'an, China). n-Hexane and ethyl acetate (EA) were purchased from Oriental Chemicals & Lab. Supplies Ltd. (Hong Kong, China). Anhydrous dichloromethane (DCM), anhydrous dimethyl sulfoxide (DMSO), anhydrous tetrahydrofuran (THF), petroleum ether (PE), hydrochloric acid (HCl), chloroform-d ($CDCl_3$), acetonirtrile-d_3 (CD_3CN), dimethylsulfoxide-d_6 (DMSO-d_6) and nile red were purchased from J&K Scientific (Hong Kong, China). Fetal bovine serum (FBS), Dulbecco′s Modified Eagle′s Medium (DMEM), penicillin/streptomycin and phosphate-buffered saline (PBS) were purchased from ThermoFisher Scientific (Hong Kong, China). All western blotting reagents were purchased from Bio-Rad Laboratories (Hong Kong, China). Dialysis bags with molecular weight cut-off (MWCO) of 1,000 and 3,500 were purchased from MYM Biological technology company limited (USA). Thin layer chromatography (TLC) plates (Aluminium sheets, silica gel 60 F254) were purchased from Labware (Hong Kong, China). All laboratory glassware was purchased from Synthware (Beijing, China). All cell-culture related consumables (Corning) were purchased from Tin Hang Technology Limited (Hong Kong, China).

Measurements: Synthesized products were purified by silica column chromatography using CombiFlash® Rf chromatography instrument (Teledyne ISCO) or preparative-scale high performance liquid chromatography (prep-HPLC) system (Agilent technologies, 1260). Chemical structures of purified products were confirmed by proton nuclear magnetic resonance (^{1}H NMR) spectroscopy on a Bruker AV300 NMR 400 MHz spectrometer. The molecular

weight (Mn) and polydispersity index (PDI) of the prepared polymers were determined by gel permeation chromatography (GPC) equipped with a waters Alliance HPLC e2685 using THF as eluent (35 ^{o}C), a 7.8 × 300 mm column, and a 2414 differential refractive index detector. The hydrodynamic diameters and surface charge of nanoparticles were measured by dynamic light scattering (DLS) using a Zetasizer Nano-ZS (Malvern instruments, UK). Transmission electron microscope (TEM) images were observed on a Hitachi HT7700 TEM. The UV-vis absorption spectra were recorded by a multi-mode microplate reader (Molecular Devices, SpectraMax M4). The X-Ray diffraction analysis (XRD) was done on a Rigaku SmartLab 9kW diffractometer with a copper rotating anode (Ka1 1.54059 Å, Ka2 1.54441 Å) rated at 200 mA/ 45 kV. Bragg-Brentano CBO incident X-ray optics was used, with a 0.5 deg incident parallel Soller slit, a 5.0 mm length limiting slit, a 0.5 deg receiving slit and a PSA Open receiving optical device, diffraction signals were filtered with a Kb Nickel filter and data were collected with a HyPix 3000 hybrid pixel array detector in 1 D mode. Diffractograms were collected with a two-theta range from 5 to 60 degrees with a 0.02 degree per step width and 5.0 degree per minute scan speed. Flow cytometry (Agilent NovoCyte Advanteon BVYG) was used to detect ROS generation *in vitro*. Laser scanning confocal microscopy (Carl Zeiss LSM 980, Germany) and flow cytometry (BD-C6, US) were conducted to analyze the cellular uptake of nanoparticles *in vitro*. The ChemiDoc Imaging System (Bio-Rad Laboratories, Hong Kong) was used for western blotting analysis. In Vivo Imaging System (IVIS, PerkinElmer, USA) was used to monitor the biodistribution of nanoparticles *in vivo*. A microcomputed tomography imaging system (Triumph X-SPECT/X-O CT, USA) was used to evaluate the bone density of mice. Flow cytometry (BD-C6, US) was performed to calculate the concentration of pro-inflammatory cytokines in serum and paws. A biochemistry analyzer (Hitachi 7100, Japan) was used to analyze levels of serum biochemical indicators.

Synthesis of ROS-nonresponsive CA-conjugated monomers: The following reagents were dissolved in 100 mL of anhydrous DCM: ditert-butyl (2S)-2-aminobutanedioate (3 g, 10.62 mmol), cinnamic acid (1.05 g, 7.08 mmol, 840 μL), HOBt (1.44 g, 10.62 mmol), EDCI (2.04 g, 10.62 mmol) and DIEA (1.75 g, 21.3 mmol, 3.71 mL). The mixture was degassed and allowed to stir at 25 ^{o}C for 16 h under nitrogen atmosphere protection. Then, the reaction solution was diluted with 100 mL of water and extracted with 100 mL of DCM three times. The combined organic layers were dried over anhydrous Na_2SO_4, filtered and concentrated under reduced pressure to give a residue. The residue was purified by silica column chromatography with PE/EA from 1/0 to 10/1(v/v) as the eluent to obtain the product as a white solid.

Next, this product (2.20 g, 5.86 mmol) was dissolved in 20 mL of dry DCM. HCl/EA (4 M, 11 mL) was added into the solution by dropwise. The mixture was allowed to react 16 h at 25 ^{o}C under nitrogen atmosphere. Finally, the residue was purified by prep-HPLC system: Column: Phenomenex C18 150*40mm*5μm; mobile phase: water (FA) -acetonitrile (ACN): B%: 5%-35%, to get a white solid, namely, nonROS-CA-monomer.

Synthesis of bromide-terminated nonROS-responsive CA-conjugated polyesters: In general, nonROS-CA-monomer (1.87 mmol, 492.28 mg) and 1,6-dibromohexane (2 mmol, 488 mg) were dissolved in 7 mL of DMSO. Then, TMG (3.74 mmol, 430 mg) was added into the mixture. After 16 h of reaction at 40 ^{o}C, the product was precipitated by a large amount of deionized water and collected after drying in a vacuum oven overnight, namely, bromide-terminated $(\text{nonROS-CA-polyester})_n$.

Synthesis of ROS-nonresponsive CA-conjugated polymeric prodrugs: The bromide-terminated $(\text{nonROS-CA-polyester})_n$ was dissolved in DMSO, and then the mixture of mPEG1000-COOH (0.286 mmol, 314 mg) and TMG (0.286 mmol, 32 mg) was introduced into the solution. The solution was allowed to stir 8 h at 40 ^{o}C. After that, the resulting product was purified by dialysis

against deionized water two days in the darkness, namely, mPEG-*b*-(nonROS-CA-polyester)$_n$-*b*-mPEG.

Preparation of Cy5.5-loaded nanoparticles: Cy5.5-loaded nanoparticles were also constructed by the nanoprecipitation method for the test of cellular uptake *in vitro* and biodistribution *in vivo*. In detail, Cy5.5 (0.5 mg) and mPEG-*b*-(ROS-CA-polyester)$_n$-*b*-mPEG (20 mg) amphiphilic copolymers were dissolved into 1 mL of THF. The mixture was pipetted into 4 mL of deionized water. Next, the solution was dialyzed against deionized water for 24 h to remove THF. After dialysis, the aqueous nanoparticle solution was allowed to pass through a 0.22-μm hydrophilic syringe filter remove the free Cy5.5.

Critical micelle concentration (CMC) determination: According to the reported method,[1] the ROS-CA-NPs stock solution (100 μg/mL) was diluted with deionized water to obtain a series of samples with concentrations from 0.2 to 100 ug/mL. Next, 5 μL of Nile red solution (1 mg/mL in THF) was pipetted into each sample tube. The mixture was incubated overnight at 200 rpm in a shaker at RT. After that, the fluorescence of sample solution was recorded by a multi-mode microplate reader (λex=535 ± 10 nm, λem= 612 ± 20 nm).

Cellular uptake: In order to investigate cellular uptake of nanoparticles, Cy5.5 was used as a fluorescence dye and entrapped into ROS-CA-NPs (Cy5.5-NPs). Cy5.5-NPs were cultured with macrophages with or without LPS activation. Laser scanning confocal microscopy (LSCM) and flow cytometry were performed to detect the fluorescence signal of Cy5.5. In general, RAW 264.7 cells at the density of 1×10^5/mL were seeded in 24-well plates or 8-well confocal chamber slides for 12 h growth. Then, the cells were stimulated by LPS (10 μg/mL) for 24 h. After stimulation, the medium was replaced with FBS-free DMEM containing Cy5.5-NPs. The system was incubated with different time points. As a control, Cy5.5-NPs were cultured with

normal cells without LPS activation. Next, all cells were washed with PBS twice. For flow cytometry study, cells were resuspended in PBS for further analysis. For confocal image analysis, cells were fixed with 4% (w/v) paraformaldehyde for 10 min at room temperature. The cells were then washed with PBS and the nuclei was stained with 4',6-diamidino-2-phenylindole (DAPI) at room temperature for 10 min, followed by washing with PBS twice for further image analysis. Data were presented by FlowJo and ZEN black 3.0, respectively.

Hemolysis test: Fresh mouse blood was extracted and stored in a heparin sodium pre-treated tube. Red blood cells (RBCs) were isolated by centrifugation at 4 ^{o}C for 10 min at 3000 rpm, and then RBCs were diluted with PBS. Nanoparticles with different concentrations were added into diluted RBCs. The mixture was incubated at 37 ^{o}C for 3 h. Equal volume of PBS or 1 % Triton X-100 incubated with RBCs served as a negative or positive control, respectively. After that, the supernatants were collected. Finally, absorbance of all samples was recorded at 540 nm using a multi-mode microplate reader. The hemolysis ratio was determined by the below formula:

$$\text{Hemolysis ratio (\%)} = \frac{\text{A sample} - \text{A negative control}}{\text{A positive control} - \text{A negative control}} * 100\%$$

A sample, A positive control and A negative control means the absorbances of each sample, 1 % Triton X-100 and PBS at 540 nm, respectively.

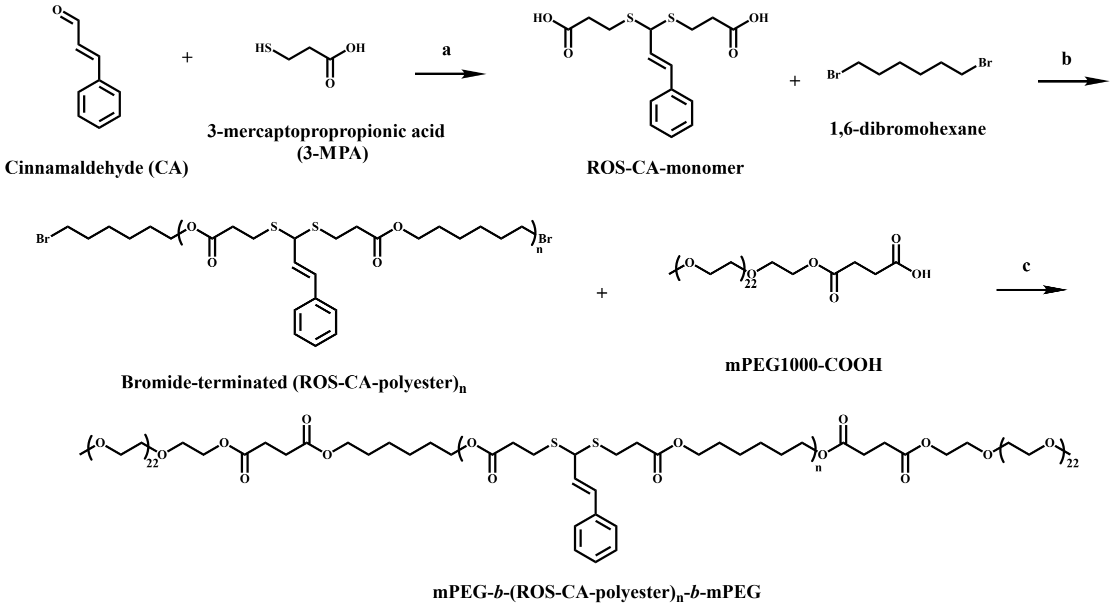

Scheme S1. Synthetic routes employed for the preparation of ROS-responsive CA-conjugated amphiphilic polymeric prodrugs. The brief reaction conditions: (a). TFA, EA; 0 °C, 24 h. (b). TMG, DMSO; 40 °C, 16 h. (c) TMG, DMSO; 40 °C, 8 h.

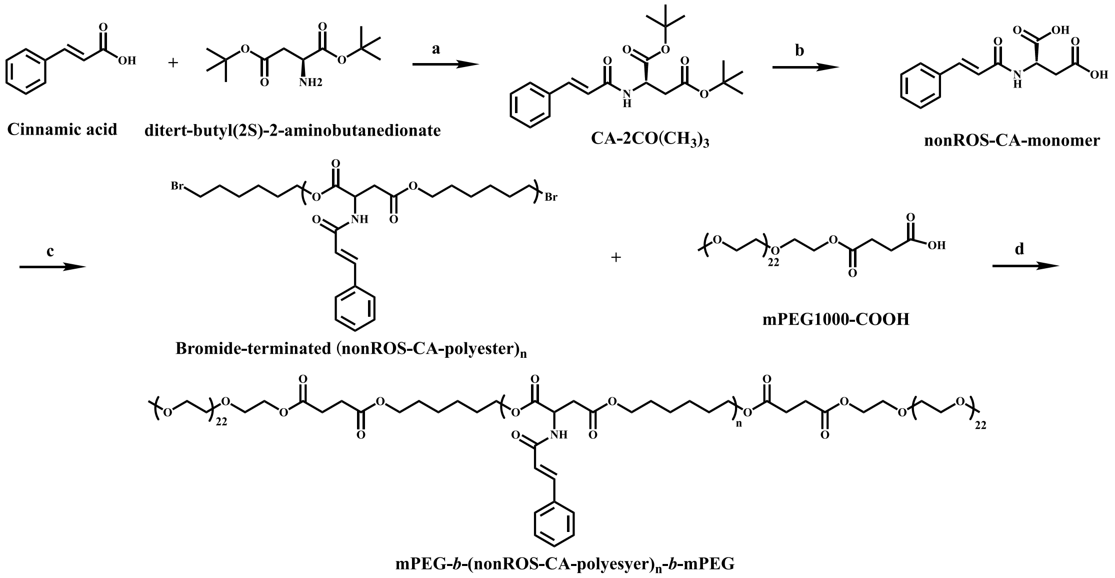

Scheme S2. Synthetic routes employed for the preparation of nonROS-responsive CA-conjugated amphiphilic polymeric prodrugs. The brief reaction conditions: (a). HOBt, EDCI, DIEA, DCM; 25 °C, 16 h. (b). HCl/EA (4 M), DCM; 25 °C, 16 h. (c). TMG, DMSO; 40 °C, 16 h. (d) TMG, DMSO; 40 °C, 8 h.

Table S1. GPC results of synthetic polyesters (Pn) and polymeric prodrugs (PPn).

	Mn[a] (Da)	PDI[a]
P1[b]	2492	1.59
P2[b]	3799	1.83
P3[b]	4323	1.92
P4[c]	3380	1.84
PP1[d]	6650	1.25
PP2[d]	8456	1.47
PP3[d]	9533	1.51
PP4[e]	5166	2.01

[a]Mn and PDI were determined by GPC in THF solvent at 35 ^{o}C using polystyrene standard.

[b]Bromide-terminated (ROS-CA-polyester)$_n$. [c]Bromide-terminated (nonROS-CA-polyester)$_n$.

[d]mPEG-*b*-(ROS-CA-polyester)$_n$-*b*-mPEG. [e]mPEG-*b*-(nonROS-CA-polyester)$_n$-*b*-mPEG.

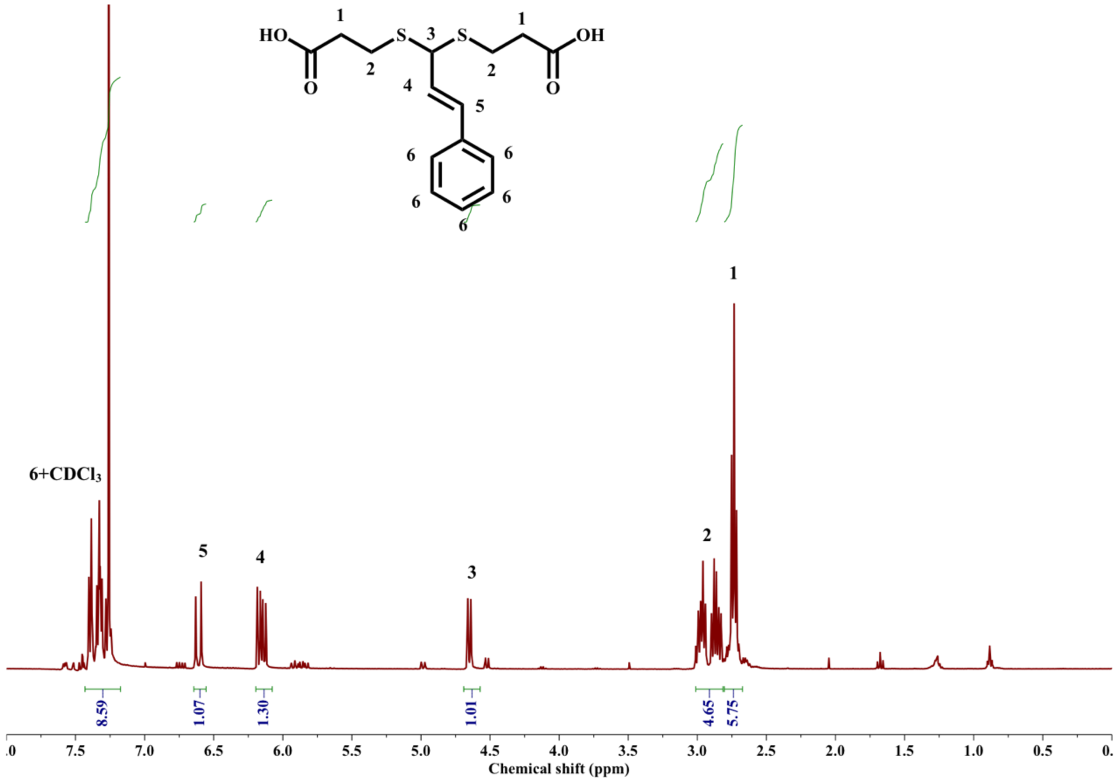

Figure S1. ^{1}H NMR spectrum recorded for ROS-CA-monomer.

A

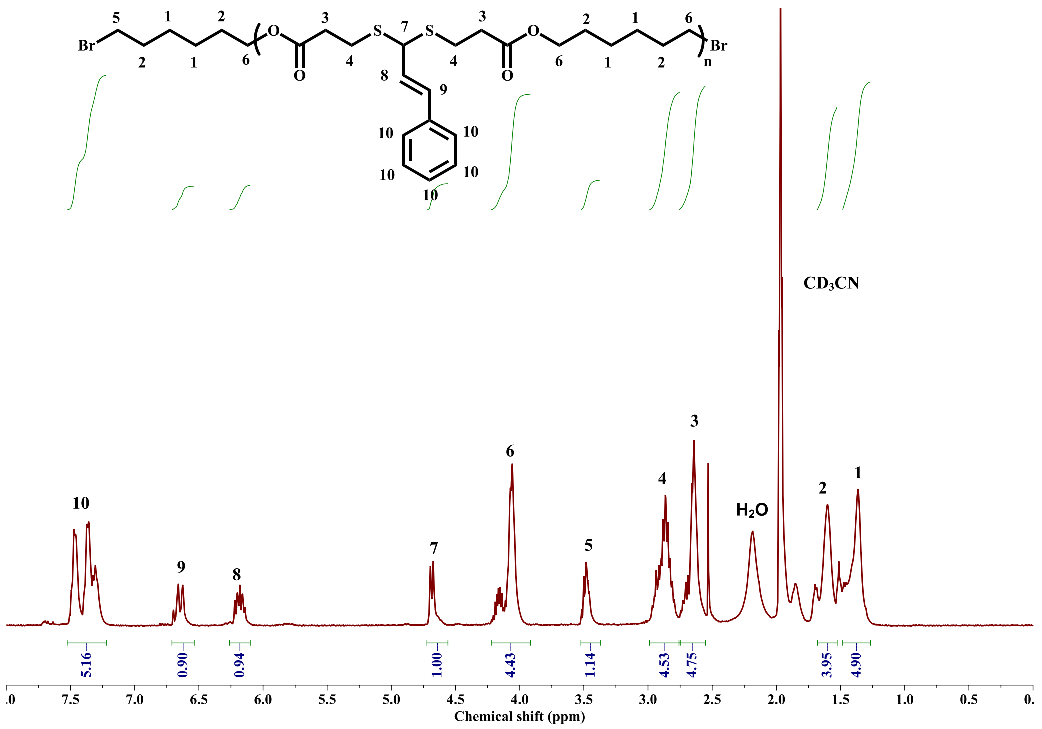

B

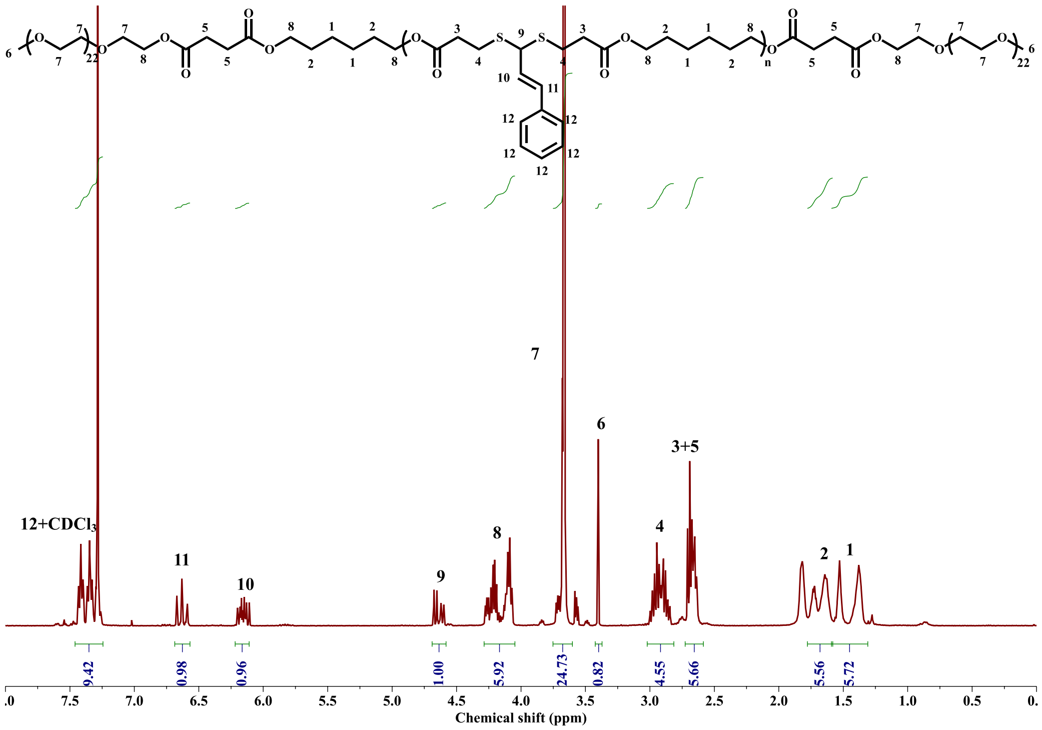

Figure S2. ^{1}H NMR spectra recorded for (A) P1: Bromide-terminated (ROS-CA-polyester)$_2$ and (B) PP1: mPEG-*b*-(ROS-CA-polyester)$_2$-*b*-mPEG. The degree of polymerization (DP) of ROS-CA-polyester (n) was determined from the ^{1}H NMR spectrum accurately (Figure S2A).

A

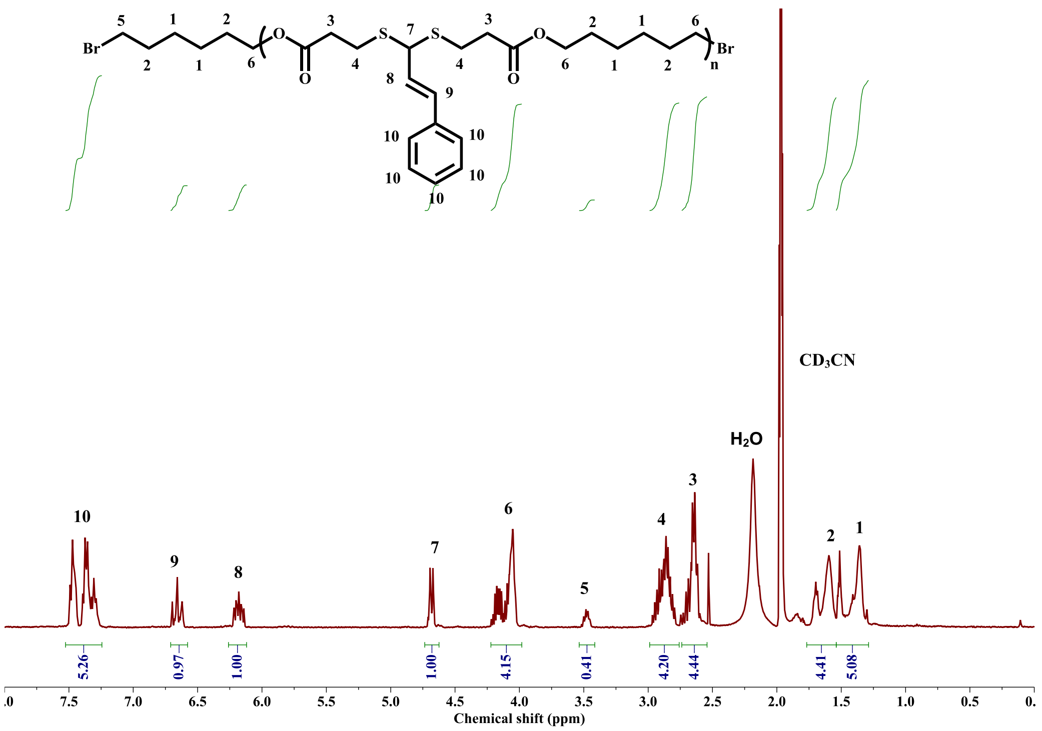

B

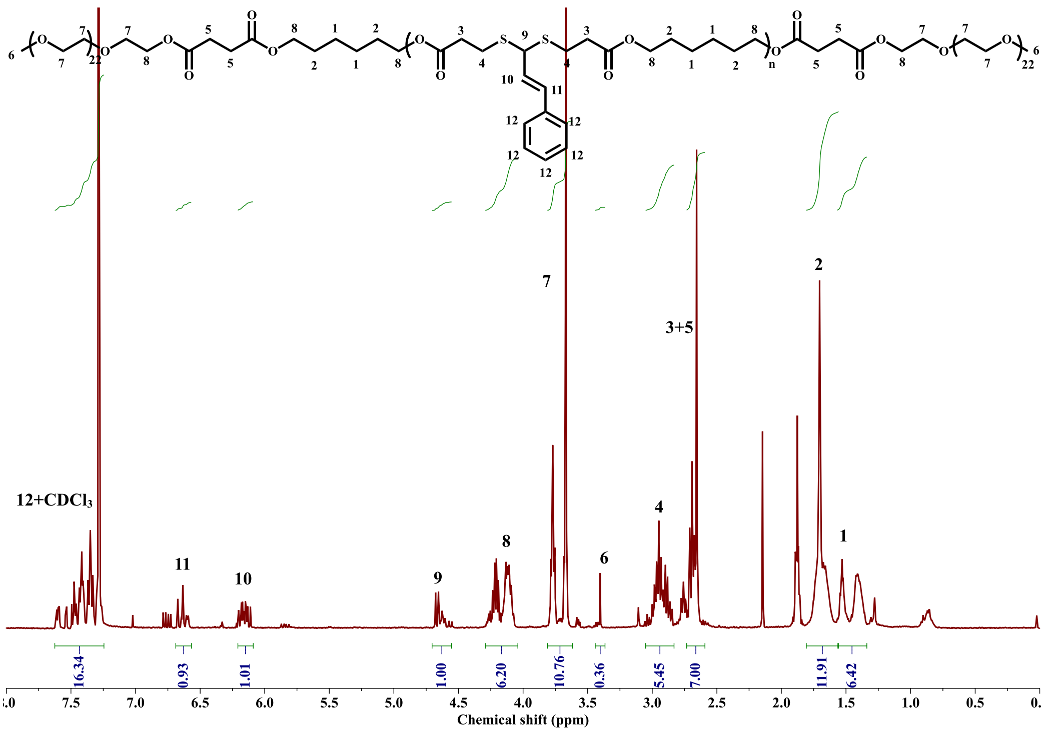

Figure S3. ^{1}H NMR spectra recorded for (A) P2: Bromide-terminated (ROS-CA-polyester)$_5$ and (B) PP2: mPEG-*b*-(ROS-CA-polyester)$_5$-*b*-mPEG. The degree of polymerization (DP) of ROS-CA-polyester (n) was determined from the ^{1}H NMR spectrum accurately (Figure S3A).

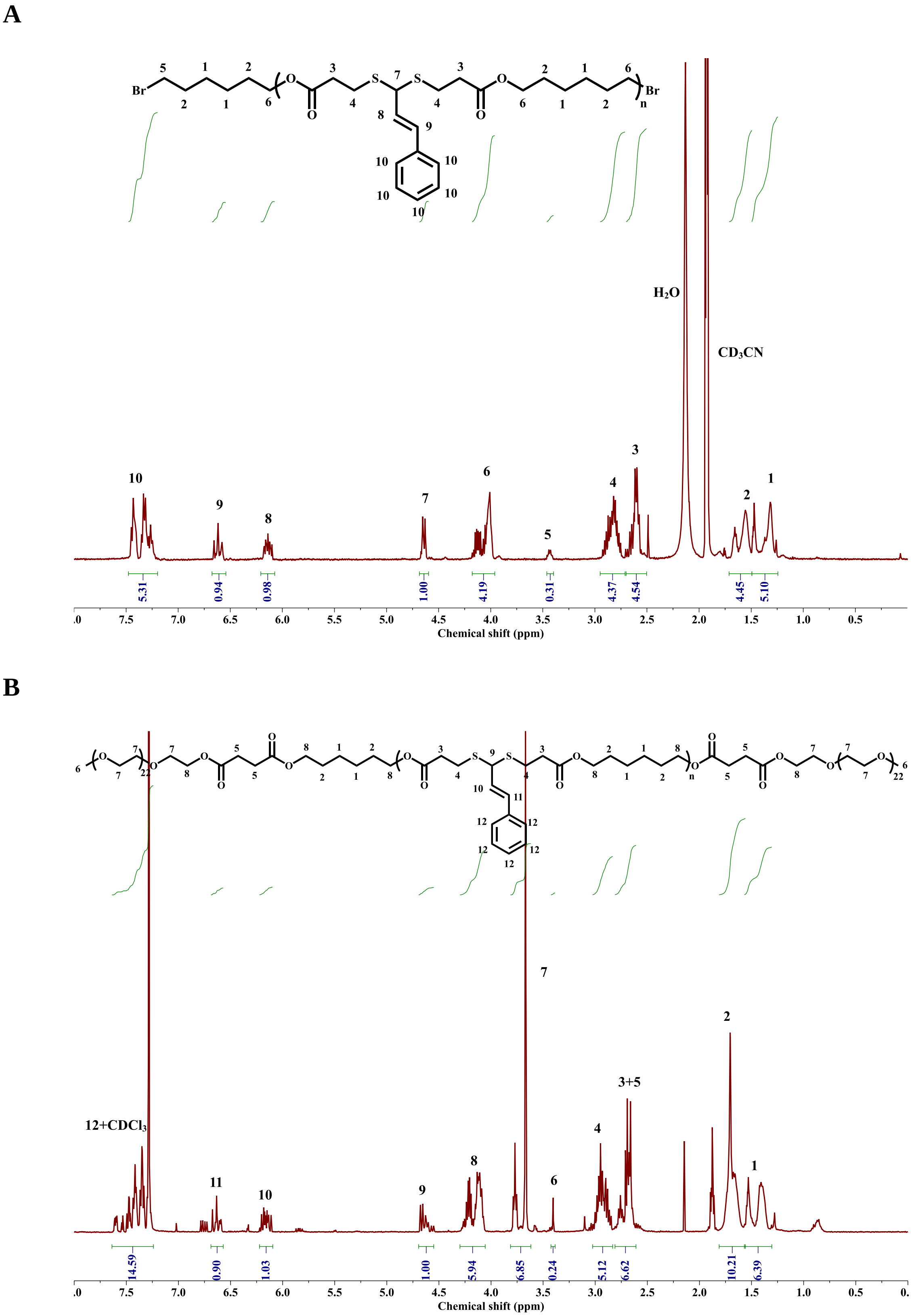

Figure S4. ^{1}H NMR spectra recorded for (A) P3: Bromide-terminated (ROS-CA-polyester)$_7$ and (B) PP3: mPEG-*b*-(ROS-CA-polyester)$_7$-*b*-mPEG. The degree of polymerization (DP) of ROS-CA-polyester (n) was determined from the ^{1}H NMR spectrum accurately (Figure S4A).

A

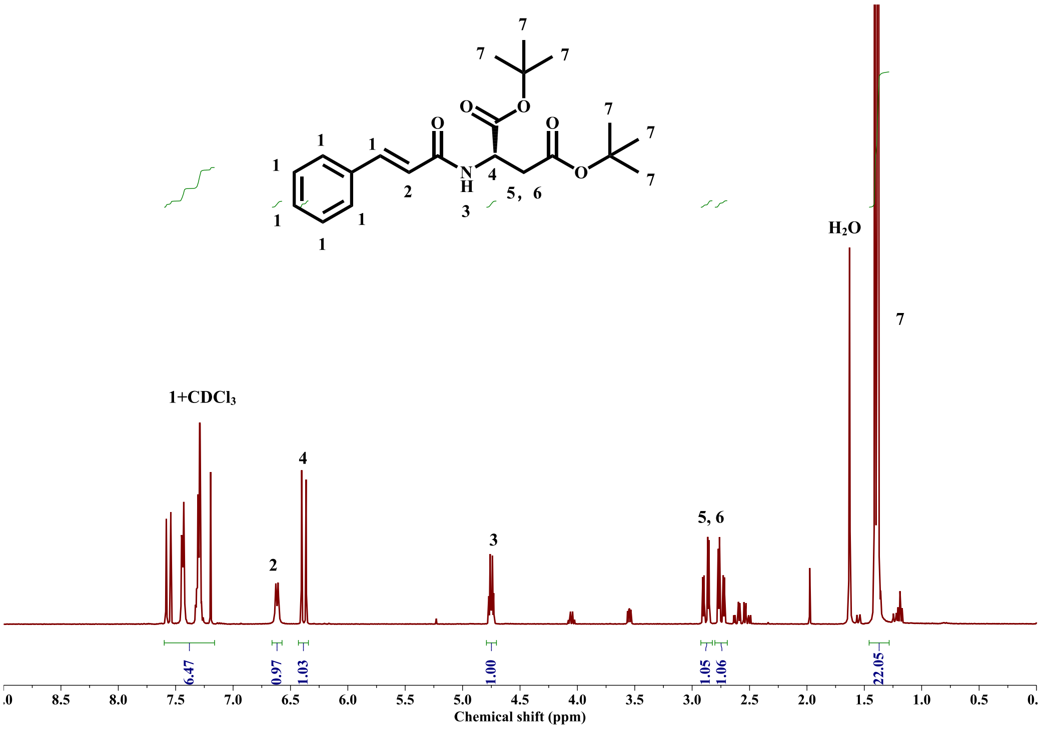

B

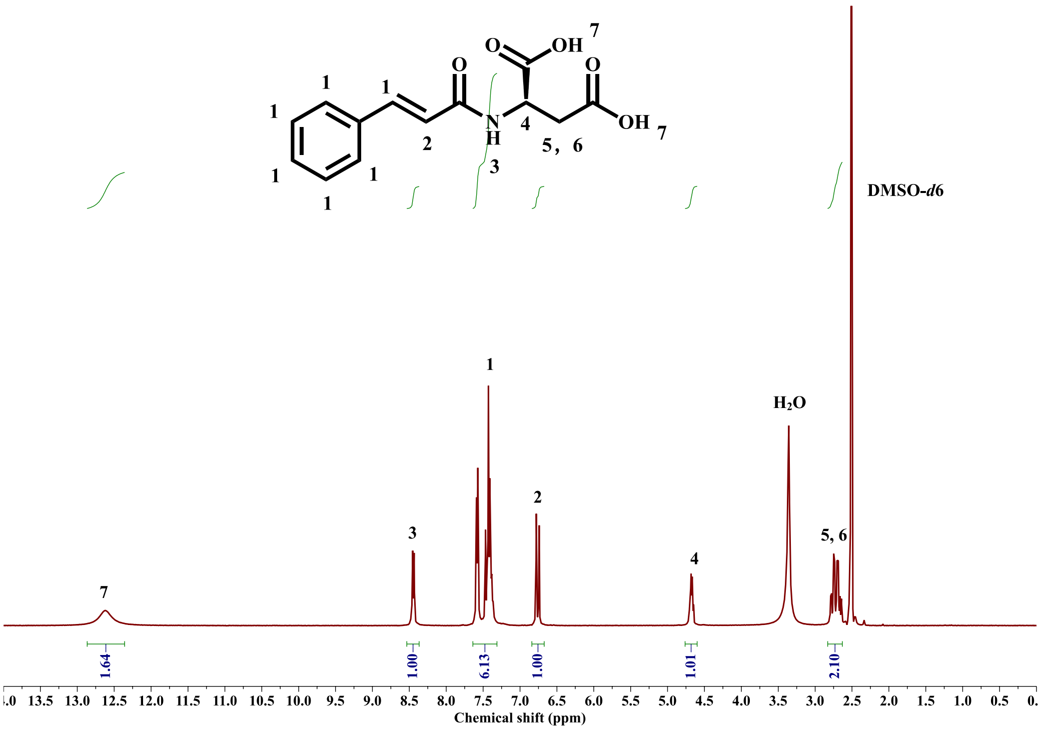

Figure S5. ^{1}H NMR spectra recorded for (A) CA-2CO(CH3)3 and (B) nonROS-CA-monomer.

A

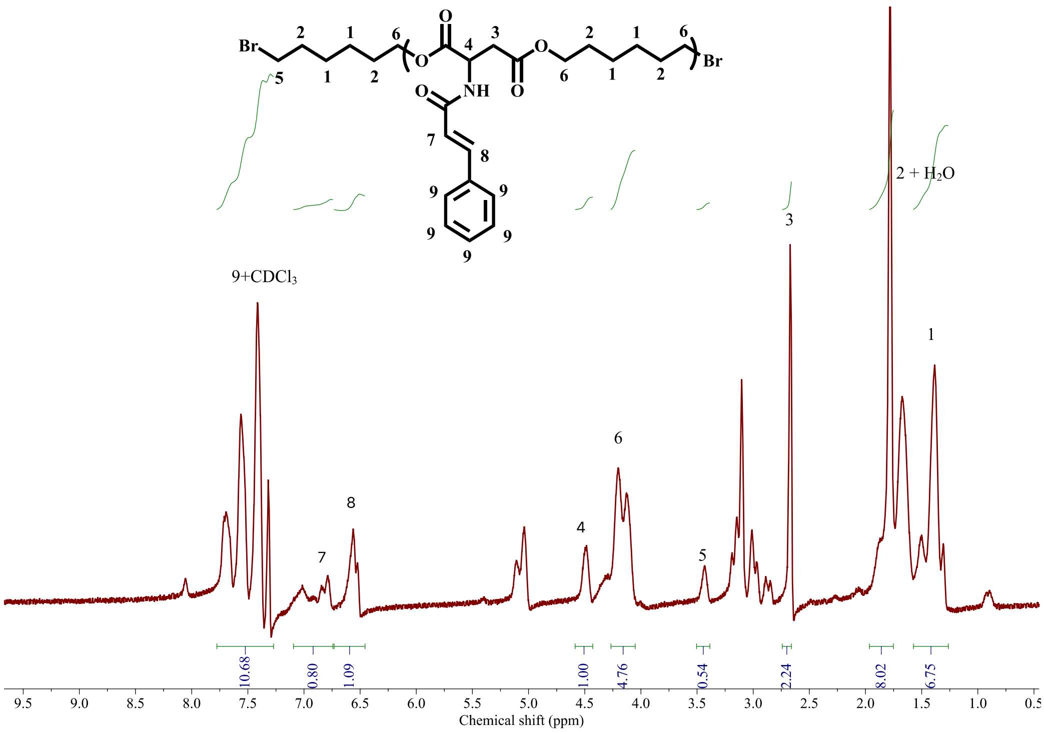

B

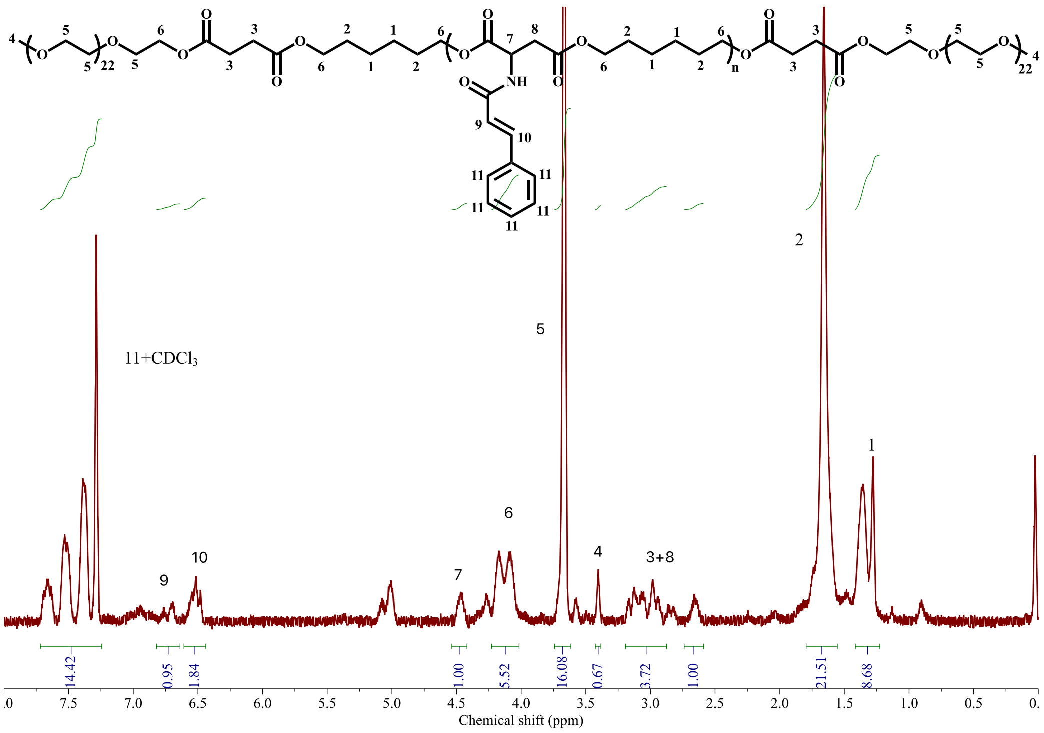

Figure S6. ^{1}H NMR spectra recorded for (A) P4: Bromide-terminated (nonROS-CA-polyester)$_4$ and (B) PP4: mPEG-*b*-(nonROS-CA-polyester)$_4$-*b*-mPEG. The degree of

polymerization (DP) of nonROS-CA-polyester (n) was determined from the ^{1}H NMR spectrum accurately (Figure S6A).

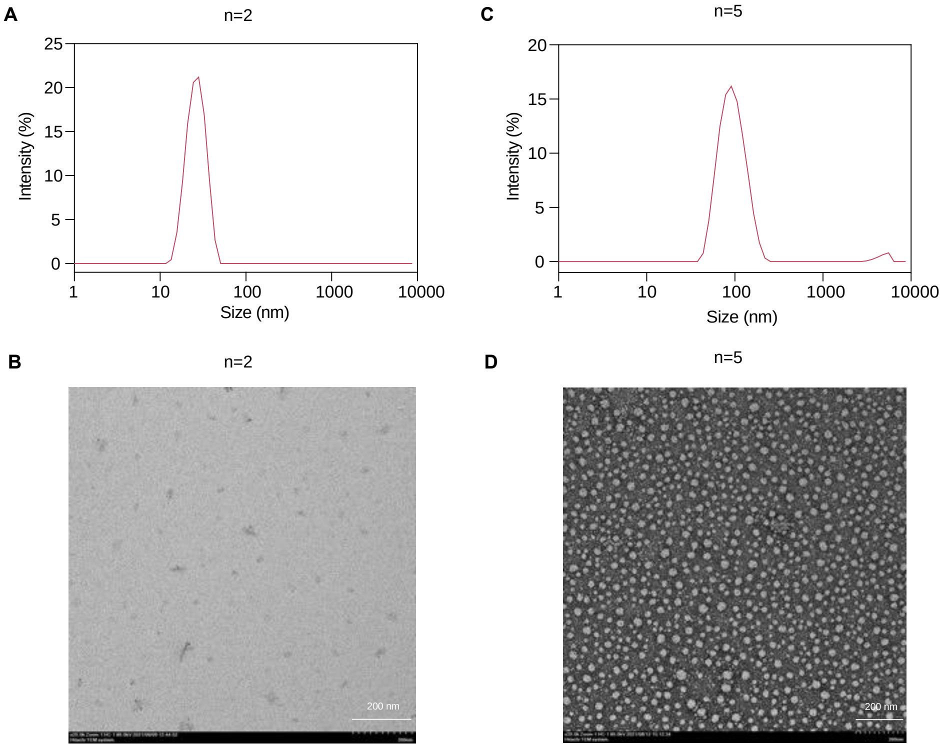

Figure S7. Characterization of ROS-responsive nanoparticles with different degree of polymerization (DPs) of CA-conjugated polyesters (n). (A) and (C) Average size distribution of ROS-responsive nanoparticles. (B) and (D) Representative TEM images of ROS-responsive nanoparticles.

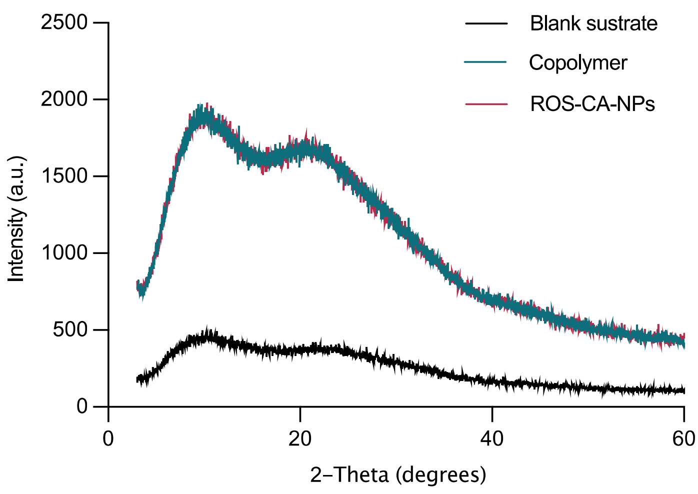

Figure S8. X-Ray diffraction pattern of ROS-CA-NPs. Copolymer: mPEG-*b*-(ROS-CA-polyester)$_7$-*b*-mPEG (PP3), which can self-assemble into nanoparticles (ROS-CA-NPs).

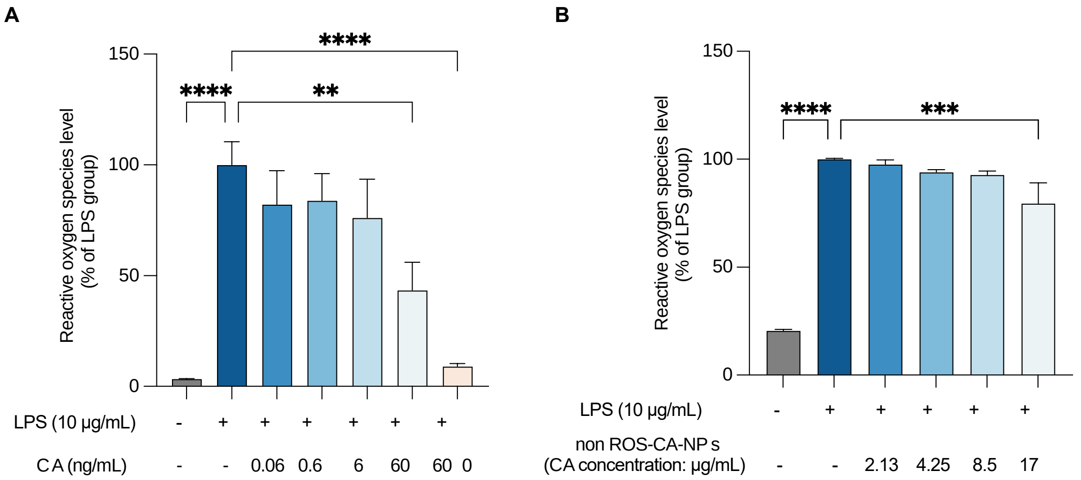

Figure S9. Levels of intracellular ROS after incubation with (A) free CA and (B) nonROS-CA-NPs quantitatively determined by flow cytometry. Data are presented as means ± SD of three independent experiments. One-way ANOVA with Tukey's multiple comparisons test, **$p < 0.01$, ***$p < 0.001$, ****$p < 0.0001$.

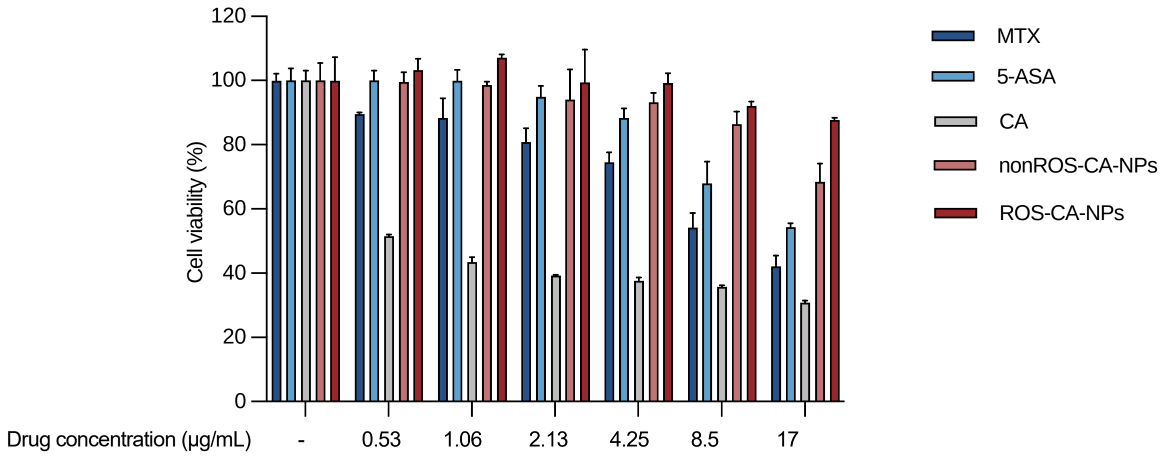

Figure S10. Cell viability was determined by the MTT assay. ROS-CA-NPs or nonROS-CA-NPs were added at different CA-equivalent concentrations. 5-ASA, 5-aminosalicylic acid; MTX, methotrexate; CA, cinnamaldehyde.

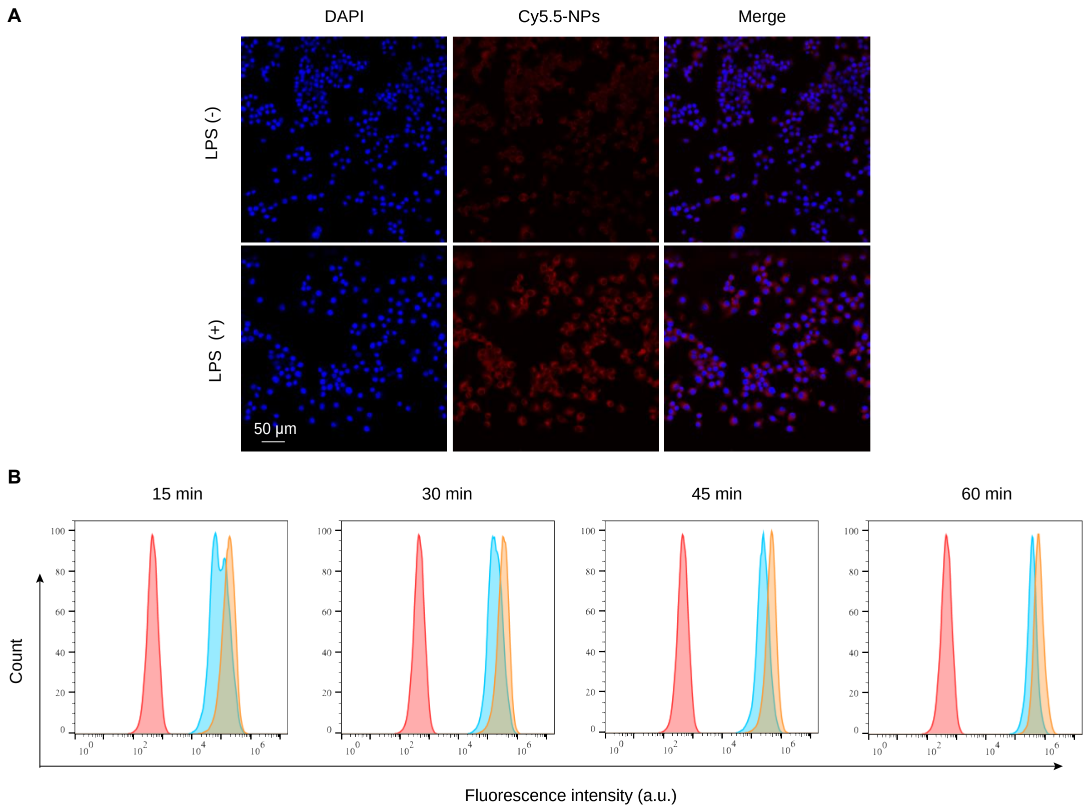

Figure S11. Cellular uptake of Cy5.5-NPs in LPS (-) and LPS (+) RAW 264.7 cells. (A) Laser scanning confocal microscopy images for evaluating the accumulation of Cy5.5-NPs in LPS (-) and LPS (+) RAW 264.7 cells. DAPI was used to label nuclei. (B) Flow cytometry analysis of Cy5.5-NPs in LPS (-) and LPS (+) RAW 264.7 cells at different incubation time intervals. (Red peak: control group of LPS (-) RAW 264.7 cells; Blue peak: Cy5.5-NPs in LPS (-) RAW 264.7 cells; Yellow peak: Cy5.5-NPs in LPS (+) RAW 264.7 cells).

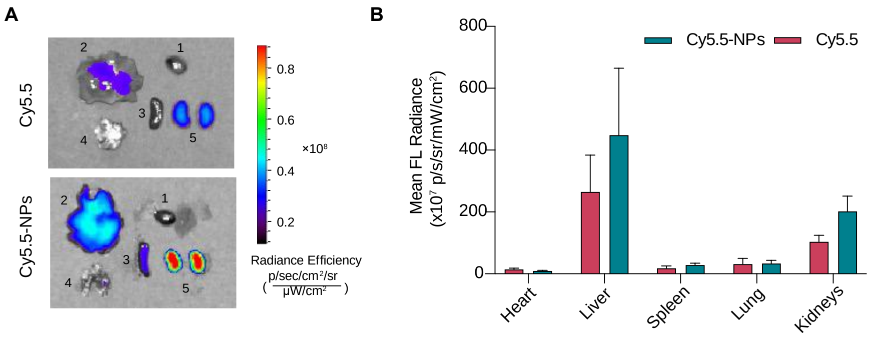

Figure S12. The *ex vivo* fluorescence intensity of Cy5.5 presented in major organs of the mice with collagen-induced arthritis (CIA). (A) Representative images of major organs collected from different groups at the end of the experiment (1. Heart, 2. Liver, 3. Spleen, 4. Lung, 5. Kidneys). (B) Region of interest (ROI) quantification of fluorescence intensity in major organs. Data are presented as mean ± SD (n = 3).

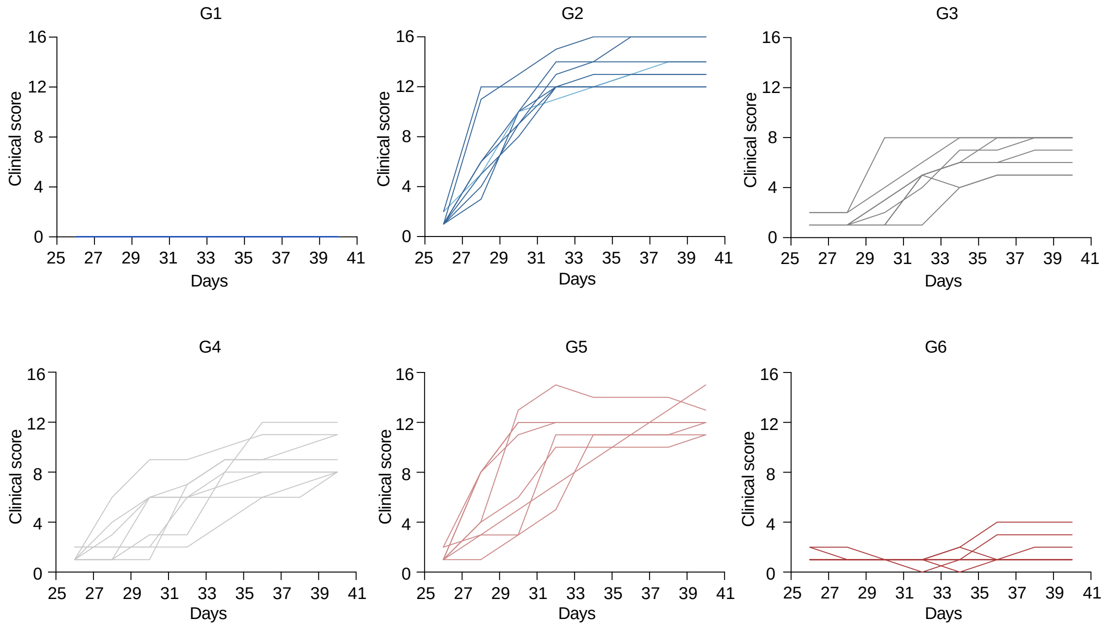

Figure S13. Clinical scores vs time curve of each mouse from different treatment groups from day 26 to day 40. Eight mice were used in each group and clinical scores were evaluated every other day. G1, phosphate-buffered saline (PBS)-treated healthy mice; G2, PBS-treated CIA mice; G3, MTX-treated CIA mice; G4, CA-treated CIA mice; G5, nonROS-CA-NP-treated CIA mice; and G6, ROS-CA-NP-treated CIA mice.

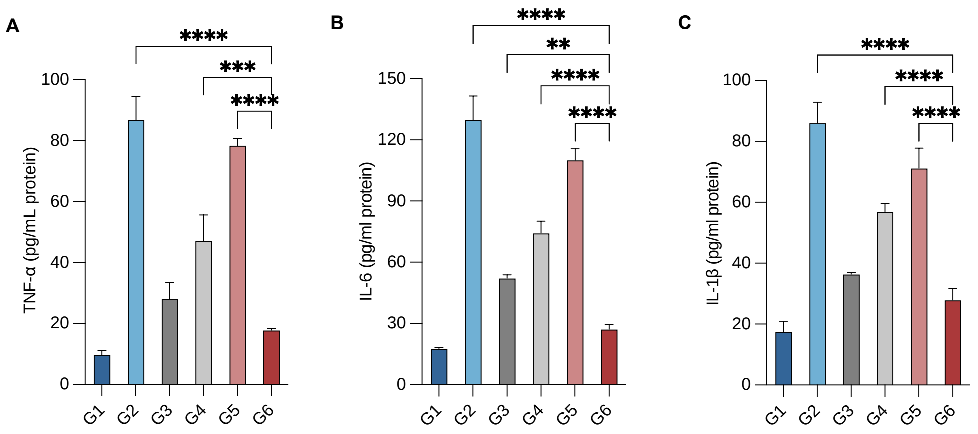

Figure S14. Concentrations of pro-inflammatory cytokines in serum. G1, phosphate-buffered saline (PBS)-treated healthy mice; G2, PBS-treated CIA mice; G3, MTX-treated CIA mice; G4, CA-treated CIA mice; G5, nonROS-CA-NP-treated CIA mice; and G6, ROS-CA-NP-treated CIA mice. Data are presented as mean ± SD (n = 3). One-way ANOVA with Tukey's multiple comparisons test, **$p < 0.01$, ***$p < 0.001$, and ****$p < 0.0001$.

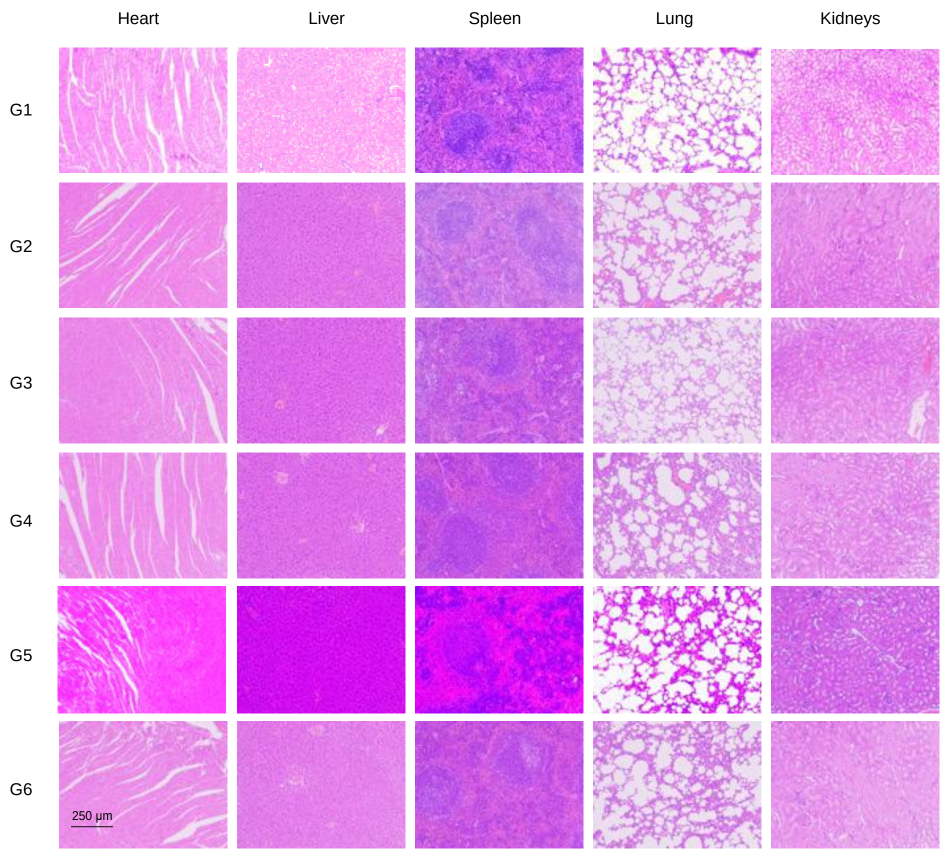

Figure S15. Hematoxylin and eosin (H&E)-stained images of major organs (heart, liver, spleen, lung, and kidneys) harvested from each treatment group. G1, phosphate-buffered saline (PBS)-treated healthy mice; G2, PBS-treated CIA mice; G3, MTX-treated CIA mice; G4, CA-treated CIA mice; G5, nonROS-CA-NP-treated CIA mice; and G6, ROS-CA-NP-treated CIA mice.

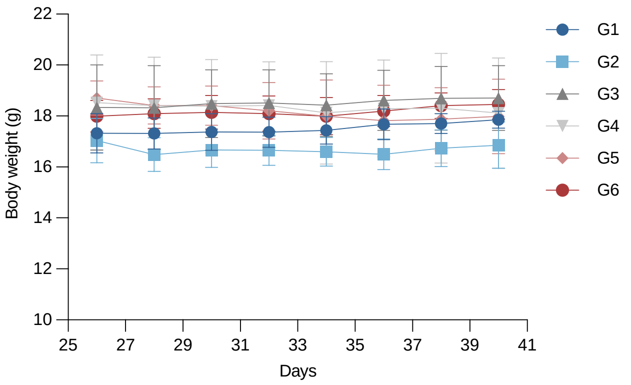

Figure S16. Body weight changes with time for G1-G6 mice during the entire treatment period. G1, phosphate-buffered saline (PBS)-treated healthy mice; G2, PBS-treated CIA mice; G3, MTX-treated CIA mice; G4, CA-treated CIA mice; G5, nonROS-CA-NP-treated CIA mice; and G6, ROS-CA-NP-treated CIA mice. Data are presented as mean ± SD (*n* = 8).

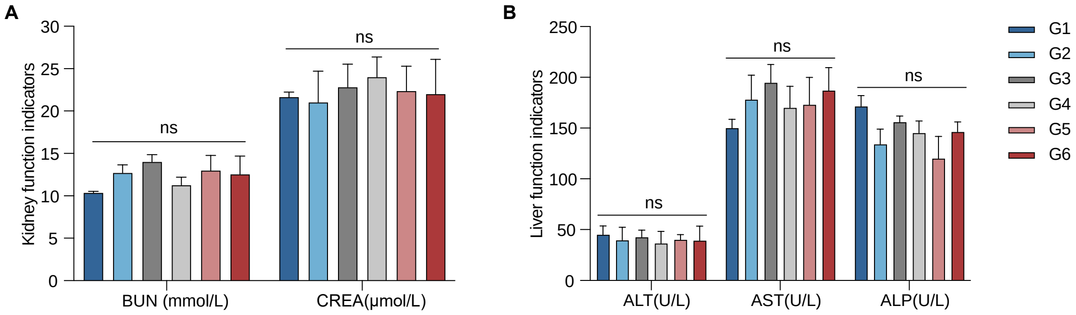

Figure S17. Serum levels of biochemical indicators of G1-G6 mice. (A) Kidney function indicators (BUN: Blood urea nitrogen, CREA: Creatinine). (B) Liver function indicators (ALT: Alanine transaminase, AST: Aspertate aminotransferase, ALP: Alkaline phosphatase). G1, phosphate-buffered saline (PBS)-treated healthy mice; G2, PBS-treated CIA mice; G3, MTX-treated CIA mice; G4, CA-treated CIA mice; G5, nonROS-CA-NP-treated CIA mice; and G6, ROS-CA-NP-treated CIA mice. Data are presented as mean ± SD (*n* = 3). One-way ANOVA with Tukey's multiple comparisons test, ns denoting no significant difference.

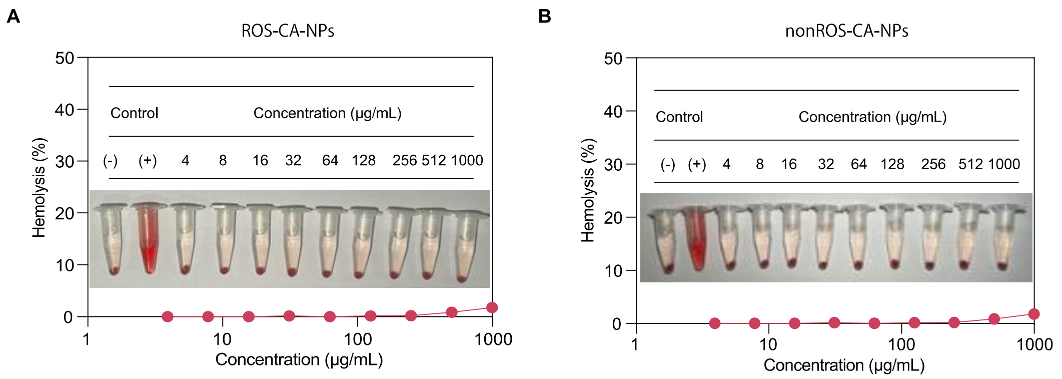

Figure S18. Hemolysis analysis of (A) ROS-CA-NPs and (B) nonROS-CA-NPs incubated with mouse red blood cells at various concentrations. PBS was used as a negative control and Triton-X100 was chosen as a positive control. Data are presented as mean ± SD (n = 3).

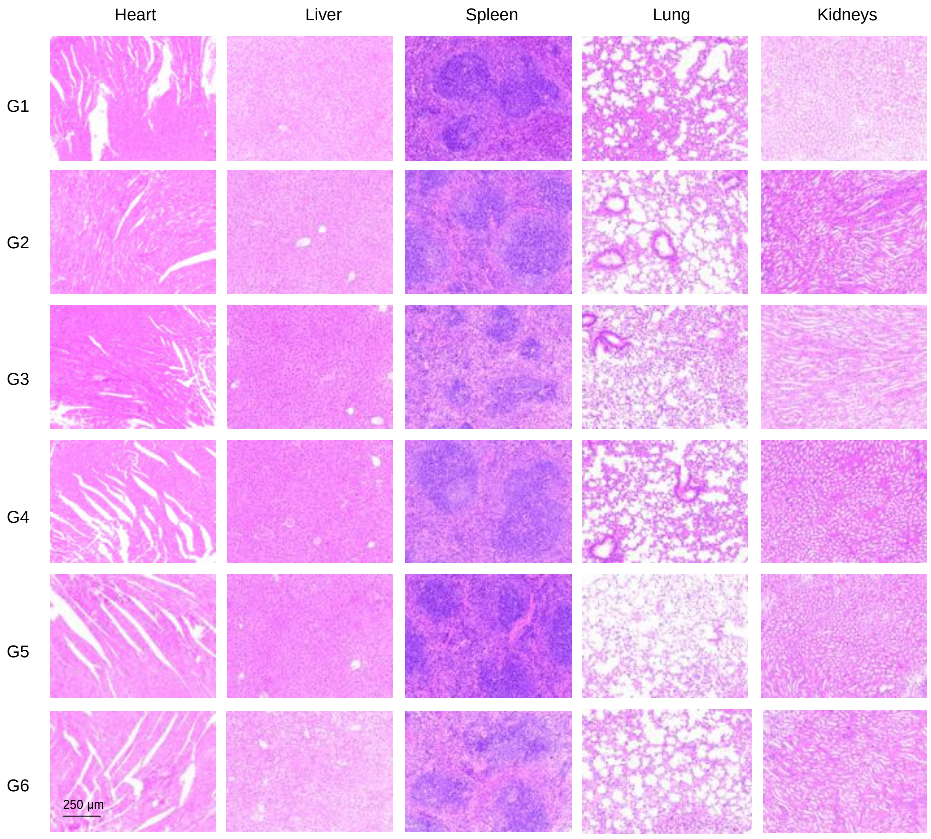

Figure S19. Hematoxylin and eosin (H&E)-stained images of major organs (heart, liver, spleen, lung, and kidneys) harvested from each treatment group. G1, PBS-treated healthy mice; G2, PBS-treated UC mice; G3, 5-ASA-treated UC mice; G4, CA-treated UC mice; G5, nonROS-CA-NP-treated UC mice; and G6, ROS-CA-NP-treated UC mice.

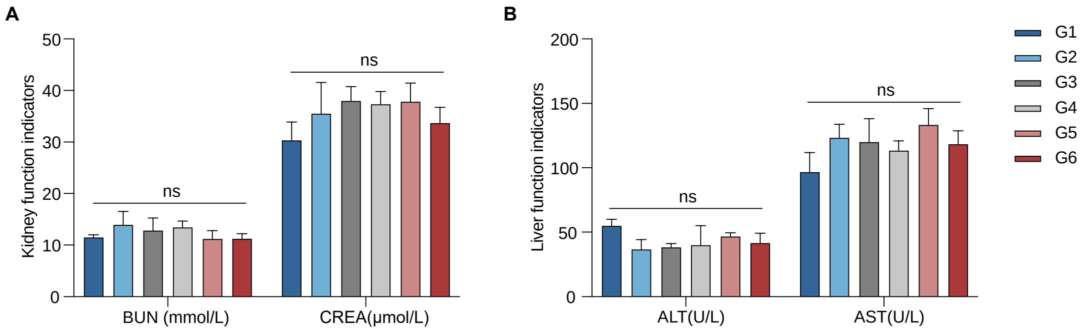

Figure S20. Serum levels of biochemical indicators of G1-G6 mice. (A) Kidney function indicators (BUN: Blood urea nitrogen, CREA: Creatinine). (B) Liver function indicators (ALT: Alanine transaminase, AST: Aspertate aminotransferase). G1, PBS-treated healthy mice; G2, PBS-treated UC mice; G3, 5-ASA-treated UC mice; G4, CA-treated UC mice; G5, nonROS-CA-NP-treated UC mice; and G6, ROS-CA-NP-treated UC mice. Data are presented as mean ± SD (n = 3). One-way ANOVA with Tukey's multiple comparisons test, ns denoting no significant difference.

Reference

[1] Y. Zhang, K. Cai, C. Li, Q. Guo, Q. Chen, X. He, L. Liu, Y. Zhang, Y. Lu, X. Chen, *Nano letters* **2018**, 18, 1908.
